# Can we assess Cancer Waiting Time targets with cancer survival? A population-based study of individually linked data from the National Cancer Waiting Times monitoring dataset in England, 2009-2013

**DOI:** 10.1371/journal.pone.0201288

**Published:** 2018-08-22

**Authors:** Chiara Di Girolamo, Sarah Walters, Carolynn Gildea, Sara Benitez Majano, Bernard Rachet, Melanie Morris

**Affiliations:** 1 Cancer Survival Group, Faculty of Epidemiology and Population Health, Department of Non-Communicable Disease Epidemiology, London School of Hygiene & Tropical Medicine, London, United Kingdom; 2 Department of Medical and Surgical Sciences, Alma Mater Studorium–University of Bologna, Bologna, Italy; 3 National Cancer Registration and Analysis Service, Public Health England, Vulcan House Steel, Sheffield, United Kingdom; National Cancer Center, JAPAN

## Abstract

**Background:**

Cancer Waiting Time targets have been integrated into successive cancer strategies as indicators of cancer care quality in England. These targets are reported in national statistics for all cancers combined, but there is mixed evidence of their benefits and it is unclear if meeting Cancer Waiting Time targets, as currently defined and published, is associated with improved survival for individual patients, and thus if survival is a good metric for judging the utility of the targets.

**Methods and findings:**

We used individually-linked data from the National Cancer Waiting Times Monitoring Dataset (CWT), the cancer registry and other routinely collected datasets. The study population consisted of all adult patients diagnosed in England (2009–2013) with colorectal (164,890), lung (171,208) or ovarian (24,545) cancer, of whom 82%, 76%, and 77%, respectively, had a CWT matching record.

The main outcome was one-year net survival for all matched patients by target attainment (‘met/not met’). The time to each type of treatment for the 31-day and 62-day targets was estimated using multivariable analyses, adjusting for age, sex, tumour stage and deprivation.

The two-week wait (TWW) from GP referral to specialist consultation and 31-day target from decision to treat to start of treatment were met for more than 95% of patients, but the 62-day target from GP referral to start of treatment was missed more often. There was little evidence of an association between meeting the TWW target and one-year net survival, but for the 31-day and 62-day targets, survival was worse for those for whom the targets were met (e.g. colorectal cancer: survival 89.1% (95%CI 88.9–89.4) for patients with 31-day target met, 96.9% (95%CI 96.1–91.7) for patients for whom it was not met). Time-to-treatment analyses showed that treatments recorded as palliative were given earlier in time, than treatments with potentially curative intent.

There are possible limitations in the accuracy of the categorisation of treatment variables which do not allow for fully distinguishing, for example, between curative and palliative intent; and it is difficult in these data to assess the appropriateness of treatment by stage. These limitations in the nature of the data do not affect the survival estimates found, but do mean that it is not possible to separate those patients for whom the times between referral, decision to treat and start of treatment could actually have an impact on the clinical outcomes. This means that the use of these survival measures to evaluate the targets would be misleading.

**Conclusions:**

Based on these individually-linked data, and for the cancers we looked at, we did not find that Cancer Waiting Time targets being met translates into improved one-year survival. Patients may benefit psychologically from limited waits which encourage timely treatment, but one-year survival is not a useful measure for evaluating Trust performance with regards to Cancer Waiting Time targets, which are not currently stratified by stage or treatment type. As such, the current composition of the data means target compliance needs further evaluation before being used for the assessment of clinical outcomes.

## Introduction

Intervals between referral for suspicion of cancer, confirmation of diagnosis and beginning of treatment are indicators of quality in cancer care: the time patients have to wait between these events have been scrutinised for many years [1–7]. Delays in waiting times have the potential both to induce worry and anxiety, which worsens patient experience [8–10], and to influence patient outcomes [11–14].

**Fig 1:**
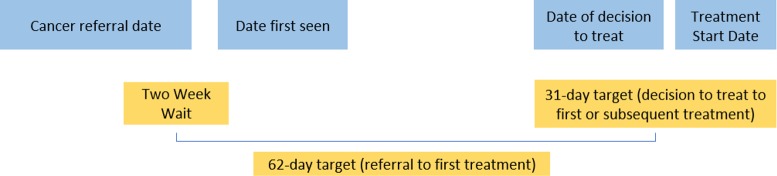
Cancer waiting times pathways and targets (adapted from cancer waiting times: A guide, version 9.0 [20]).

Waiting time standards were introduced in England in the late 1990s to improve timely diagnosis of breast cancer [15]. Subsequently, the National Health Service (NHS) Cancer Plan, published in 2000, included a series of targets to reduce waiting times to diagnosis and treatment for all cancers [16]. These included a maximum two-week wait (TWW) between an urgent referral for a suspicion of cancer from a general practitioner (GP) to being seen by a specialist, a maximum 62 days from the referral to the start of the first treatment, and a maximum 31 days from the decision taken to treat a patient to the start of the first treatment, irrespective of the route to diagnosis the patient went through (Fig 1). The Cancer Waiting Time targets were slightly revised in the 2007 Cancer Reform Strategy [17] and retained in England’s updated 2011 and 2015 national cancer strategies [18, 19]. The 2015 strategy also proposed a new 28-day target in which to confirm or rule out a cancer diagnosis following urgent referral with a suspicion of cancer.

For each waiting time target, NHS England sets operational standards for the Clinical Commissioning Groups that state the expected performance thresholds. The current official operational standards are to meet the targets for 93% of patients for whom the TWW target is applicable, 85% for the 62-day and 96% for the 31-day targets. Amendments to the official time interval calculations are allowed if factors outside the hospital’s control were involved [21]. National Cancer Waiting Time statistics, including the proportion of patients seen within target times, have been published by NHS England as quarterly national statistics since 2013–2014 [22] and previously by the Department of Health. Adherence to the 62-day target has fallen below the operational standard every quarter since January-March 2014 [23–25] and has generally decreased over this period too.

Research has evaluated the association of waiting time intervals with the outcomes of cancer patients [4, 26–30]. So far, however, little is known about the association of patients’ survival from cancer with meeting the official waiting time targets in England, while there is a general temptation to use these published targets in association with cancer survival. In this study, we use the National Cancer Waiting Times Monitoring Dataset (CWT), used for the official statistics and for system performance assessment. We focus on three cancer sites, examining whether these patients were seen or treated within the target time, and their time to treatment. As cancer survival is often used as a metric to judge the performance of the healthcare system and the quality of cancer care in particular, we assess whether there is an association between meeting waiting time targets, as currently available to the policy-makers, and individual patients’ cancer survival, and measure the time to different types of treatments in order to understand the patterns found.

## Methods

### Ethics statement

This research does not use human participants, but routinely collected data. The Cancer Survival Group obtained ethical and statutory approvals to use these data from the National Research Ethics Service Committee London–Camden & Islington on 28 May 2013 (Research Ethics Committee reference 13/LO/0610, confirmed on 29 January 2015). All data are anonymised and the researchers had no access to personally identifiable data.

### Data sources and study population

All adults aged 15–99 years who were diagnosed in England between 2009 and 2013 with a primary, invasive and malignant colorectal (ICD10: C18-C20 and C21.8), non-small cell lung (ICD10: C34, ICD-O-3: 8046/3), or ovarian cancer (ICD10: C56 to C57.7) were eligible for inclusion in the analysis. Synchronous tumours and other primaries at the same site were excluded during the cleaning process, described elsewhere [31]. Individual demographic and clinical information on these patients was obtained by linking the cancer registration data accessed through the Cancer Analysis System (CAS) at the National Cancer Registration and Analysis Service (NCRAS) and data from the National Cancer Waiting Times Monitoring Dataset (CWT) from NHS England. Additional information was available for colorectal and lung cancers from the National Bowel Cancer Audit Project and the National Lung Cancer Audit database, respectively.

The data include information on date of birth, sex, vital status, follow-up dates (available up to 31 December 2014), and an ecological deprivation quintile (1 = least deprived, 5 = most deprived) derived from the income domain of the Index of Multiple Deprivation for England [32]. Age was treated as a categorical variable and split into groups (15–44, 45–54, 55–64, 65–74, and 75+). Stage at diagnosis was obtained by drawing upon multiple sources of data (from CAS, and audit data where available), and hierarchically combining individual information [33] on tumour size (T), number of nodes involved (N), and presence of metastasis (M) to assign a summary stage category [34].

CWT includes a range of information (e.g. relevant dates, details of the source of referral, broad categories of treatment types) for cancer patients cared for in the NHS, including those who refused treatment or who were deemed not to need any anti-cancer treatment at that point but likely to need it in the future (active monitoring) [20]. CWT does not record information on those patients who die before the treatment can commence or who are diagnosed and/or treated in the private sector, including those who receive specialist palliative care in a non-NHS setting. We summarised the treatment information available in CWT into six categories based on the nature of treatment: surgery, anti-cancer drug regimen, radiotherapy, palliative treatment, active monitoring and treatment declined (S1 Table). ‘Other therapies’ (a CWT category) were omitted when stratifying results by treatment as they make up less than 0.5% of treatments for each cancer site, and are a group of varied treatments.

When a single tumour had several CWT records corresponding to different treatments, we gave priority to the record of the first treatment (as defined by a specific variable in CWT, and in accordance with practice at Public Health England), and where there was more than one record referring to the first treatment, we selected the record according to the proximity of the dates of treatment and diagnosis. For all patients linked to CWT, we calculated whether the 31-day target from decision to treat (i.e. “the date that a patient agrees to a treatment plan” [20]) to the start date of first treatment (‘31-day target’ hereafter) had been met. For the subset of patients referred through the urgent GP referral route, we calculate the following: the time between urgent GP referral for suspected cancer and the date the patient was first seen by a specialist (two-week wait target, ‘TWW’ hereafter); and the time between urgent GP referral for suspected cancer and the start date of first treatment (‘62-day target’ hereafter).

### Statistical analysis

Descriptive and survival analyses were conducted for each target and cancer site. Patients were classified according to whether the specified target was ‘not met’ or ‘met’. Analyses relating to the 31-day target and the 62-day target were restricted to patients who survived at least 90 days after cancer diagnosis to avoid the ‘time-guarantee’ bias caused by the time-dependent definition of the exposure [35]. Thus, patients who died before the target time was reached were excluded from analyses. Further, our 90-day cut-off selected a group of patients for whom timely treatment was more likely to make a real difference to their prognosis because it excluded the sickest patients who might have died irrespective of the timeliness of receiving treatment.

Net survival is the probability of patients surviving from cancer after adjusting for other competing causes of death. Mortality hazard from these competing causes was obtained using English population life tables stratified by age, sex, calendar year, deprivation and region [36]. We used the Pohar-Perme estimator [37] which is a non-parametric, consistent estimator of net survival, measured from the cancer registry date of diagnosis. One-year net survival, by target attainment, was estimated and stratified by age and tumour stage.

Additional survival analyses were also performed, by finer waiting time intervals from decision to treat to first treatment (≤10 days, 11–31 days, 32–45 days and >45 days), and stratified by stage. Preliminary analyses were also undertaken by certain types of treatments to investigate if this would clarify the outcomes: results were however similar and therefore not included. We also performed the survival analyses by target attainment, including all patients, to demonstrate survival for the whole cohort including those who died within 90 days; these results are presented in S1 Fig for reference.

A univariate Kaplan-Meier analysis and then a multivariable Cox proportional hazards regression were used to analyse the time to the start of treatment. This time was measured from referral for the 62-day target pathway and from the decision to treat date for the 31-day target pathway. The Cox regression analyses included adjustment for age, sex, stage and deprivation, and were stratified by treatment type. The results of the time-to-treatment analyses are presented as the proportion of patients who had received treatment by each point in time.

All analyses were performed using Stata 14.0.

## Results

**Fig 2:**
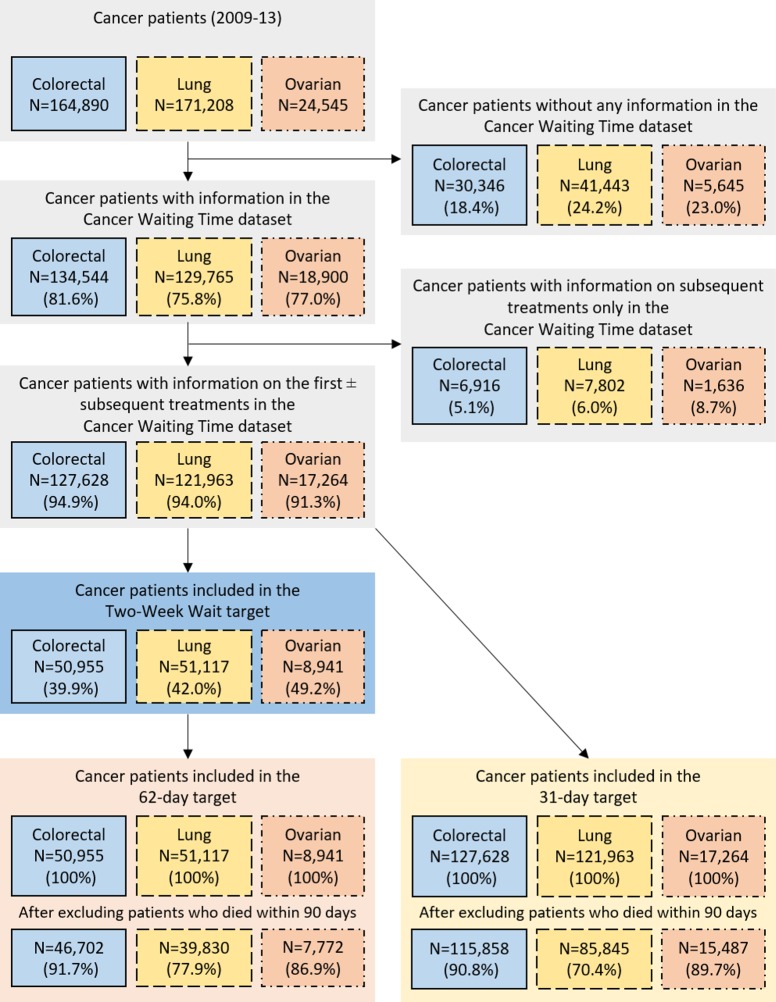
Numbers and percentages of cancer patients who were recorded in the cancer waiting times dataset and for whom the waiting time targets can be calculated, England, 2009–2013 (percentages of patients remaining calculated from the total number of the cell above).

During 2009–2013, 164,890 colorectal cancer, 171,208 non-small cell lung cancer (lung cancer hereafter), and 24,545 ovarian cancer patients were registered in England. The percentages of these patients who had information in CWT on the first and/or subsequent treatments were 82%, 76%, and 77%, respectively. Patients without CWT information or who only had information on subsequent treatments were excluded from the analyses, leaving 127,628 colorectal, 121,963 lung and 17,264 ovarian cancer patients (Fig 2).

### Association of meeting waiting time targets with cancer survival

**Fig 3:**
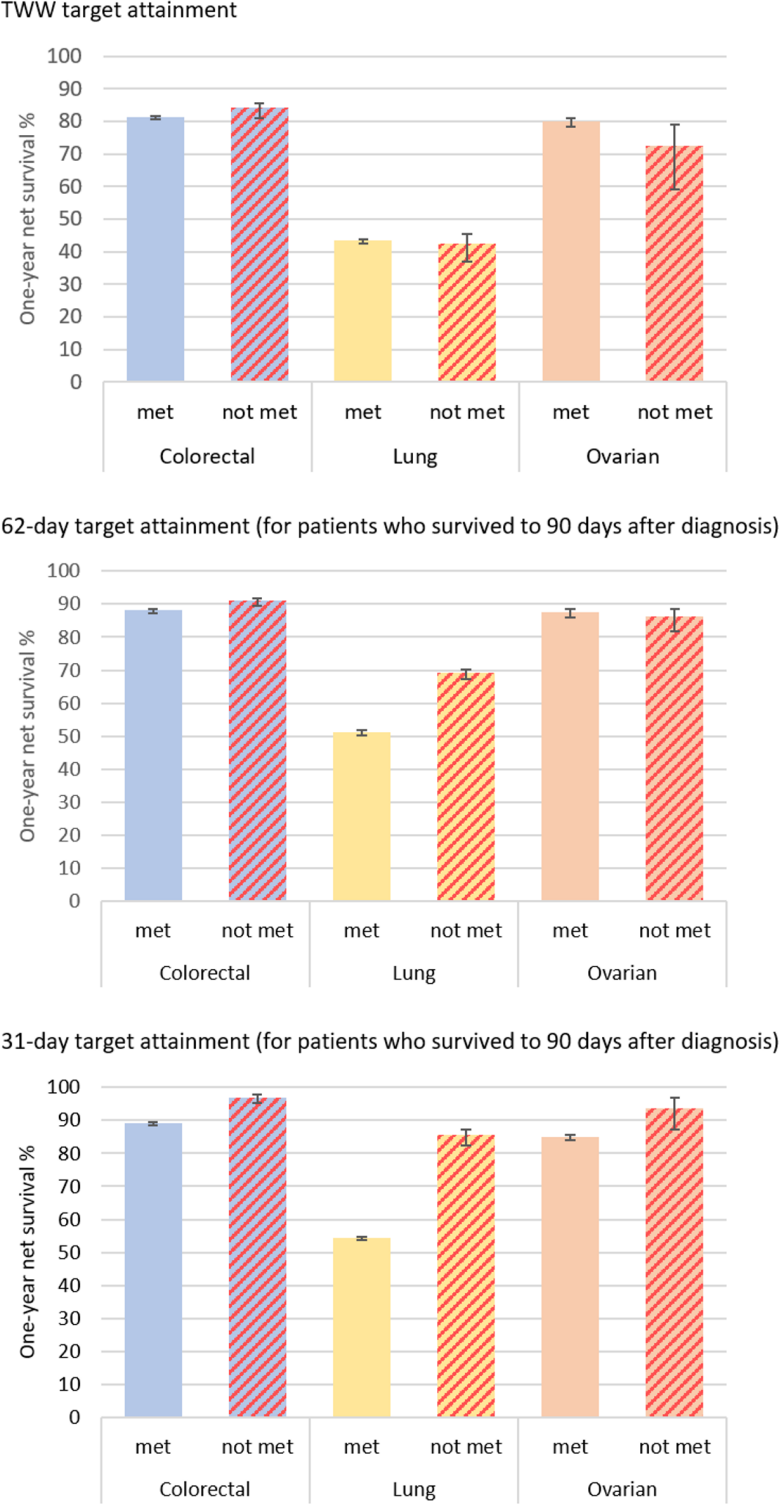
One-year net survival by target attainment for each cancer site.

There was very little difference in one-year net survival by attainment of the TWW target for any of the three cancer sites (Fig 3). Differences in net survival by 62-day target attainment (conditional on surviving more than 90 days) were seen for lung cancer and, to a lesser extent, for colorectal cancer patients, but not for ovarian cancer patients, with those having the target met more likely to have poorer survival (as shown in Fig 3). The net survival analyses by attainment of the 31-day target show that colorectal, lung and ovarian cancer patients who received their first course of treatment later than 31 days tended to have higher one-year survival than those who were treated shortly after diagnosis (Fig 3). The survival curves for one year, for the three cancers and by the 31-day and 62-day targets, are reported for the whole cohort in S1 Fig. These show the same patterns, however survival is generally worse, as those who died within 90 days are included.

### Characteristics of patients by target attainment

The TWW and 31-day targets were met for most patients while the 62-day target was missed for 26.2%, 25.2% and 14.5% of patients with colorectal, lung and ovarian cancer respectively.

The TWW was met for more than 95% of patients for all cancers and in all subgroups, although the proportion of lung and ovarian cancer patients for whom the target was missed tended to increase with age (Table 1). The 62-day target was met for between 60% and 92% of those who survived more than 90 days, depending on the cancer site and subgroup. The proportion of patients for whom the target was missed increased with age.

**Table 1:** Target attainment by patient characteristics for each waiting time target and cancer site, England, 2009–2013.

		**Colorectal cancer**		**Lung cancer**		**Ovarian cancer**	
***TWW pathway*: *all patients***		**met, N (%)**	**not met, N (%)**	**met, N (%)**	**not met, N (%)**	**met, N (%)**	**not met, N (%)**
**Total**		**48,441 (95.1)**	**2,514 (4.9)**	**49,780 (97.4)**	**1,337 (2.6)**	**8,306 (97.8)**	**185 (2.2)**
**Age groups**	15–44	721 (94.7)	40 (5.3)	424 (98.6)	6 (1.4)	474 (98.5)	7 (1.5)
	45–54	3,168 (94.5)	184 (5.5)	2,703 (97.3)	74 (2.7)	1,141 (98.2)	21 (1.8)
	55–64	8,584 (94.9)	464 (5.1)	10,375 (97.8)	228 (2.2)	2,144 (98.3)	38 (1.7)
	65–74	13,843 (95.1)	714 (4.9)	17,905 (97.6)	443 (2.4)	2,495 (97.6)	61 (2.4)
	75+	22,125 (95.2)	1,112 (4.8)	18,373 (96.9)	586 (3.1)	2,052 (97.3)	58 (2.7)
**Stage at diagnosis**	I	5,078 (94.3)	305 (5.7)	5,983 (97.2)	175 (2.8)	1,697 (97.6)	41 (2.4)
	II	9,744 (94.9)	529 (5.1)	4,344 (97.4)	116 (2.6)	502 (97.3)	14 (2.7)
	III	10,423 (95.3)	509 (4.7)	14,050 (97.2)	403 (2.8)	2,472 (98.3)	44 (1.7)
	IV	10,677 (95.6)	408 (4.6)	21,923 (97.6)	537 (2.4)	1,434 (97.8)	32 (2.2)
	Missing	12,519 (94.9)	674 (5.1)	3,480 (97.0)	106 (3.0)	2,201 (97.6)	54 (2.4)
**Deprivation quintile**	1—least deprived	10,288 (95.4)	497 (4.6)	7,064 (98.0)	146 (2.0)	1,800 (97.7)	42 (2.3)
	2	10,835 (95.2)	549 (4.8)	8,803 (97.6)	219 (2.4)	1,922 (97.8)	43 (2.2)
	3	10,437 (95.1)	542 (4.9)	10,048 (97.4)	272 (2.6)	1,804 (97.9)	39 (2.1)
	4	9,651 (95.2)	487 (4.8)	11,829 (97.2)	341 (2.8)	1,566 (97.3)	43 (2.7)
	5—most deprived	7,230 (94.3)	439 (5.7)	12,036 (97.1)	359 (2.9)	1,214 (98.5)	18 (1.5)
**Sex**	Female	20,742 (94.9)	1,106 (5.1)	22,297 (97.3)	613 (2.7)	-	-
	Male	27,699 (95.2)	1,408 (4.8)	27,483 (97.4)	724 (2.6)	-	-
**Tumour site**	Colon	27,038 (95.1)	1,392 (4.9)	-	-	-	-
	Rectum	21,403 (95.0)	1,122 (5.0)	-	-	-	-
***62-day target*: *patients who survived at least 90 days*** ^a^							
	**Total**	**34,154 (73.1)**	**12,548 (26.9)**	**29,796 (74.8)**	**10,034 (25.2)**	**6,646 (85.5)**	**1,126 (14.5)**
**Age groups**	15–44	594 (80.2)	147 (19.8)	308 (80.4)	75 (19.6)	430 (92.1)	37 (7.9)
	45–54	2,565 (79.1)	677 (20.9)	1,890 (79.3)	493 (20.7)	1,036 (90.5)	109 (9.5)
	55–64	6,597 (76.7)	2,008 (23.3)	6,718 (76.3)	2,091 (23.7)	1,859 (88.5)	242 (11.5)
	65–74	9,952 (73.5)	3,589 (26.5)	10,616 (72.6)	3,997 (27.4)	1,993 (84.0)	381 (16.0)
	75+	14,446 (70.2)	6,127 (29.8)	10,264 (75.2)	3,378 (24.8)	1,328 (78.8)	357 (21.2)
**Stage at diagnosis**	I	3,749 (70.6)	1,560 (29.4)	3,591 (60.5)	2,341 (39.5)	1,527 (88.6)	196 (11.4)
	II	7,301 (73.5)	2,633 (26.5)	2,573 (61.3)	1,627 (38.7)	433 (85.7)	72 (14.3)
	III	7,678 (73.0)	2,844 (27.0)	9,322 (74.6)	3,173 (25.4)	2,005 (84.7)	363 (15.3)
	IV	6,734 (75.4)	2,198 (24.6)	12,295 (83.8)	2,370 (16.2)	1,041 (83.5)	206 (16.5)
	Missing	8,692 (72.4)	3,313 (27.6)	2,015 (79.4)	523 (20.6)	1,640 (85.0)	289 (15.0)
		**Colorectal cancer**		**Lung cancer**		**Ovarian cancer**	
***62-day target*: *patients who survived at least 90 days*** ^a^		**met, N (%)**	**not met, N (%)**	**met, N (%)**	**not met, N (%)**	**met, N (%)**	**not met, N (%)**
**Deprivation quintile**	1—least deprived	7,331 (73.4)	2,662 (26.6)	4,337 (76.2)	1,353 (23.8)	1,431 (84.2)	268 (15.8)
	2	7,643 (73.0)	2,826 (27.0)	5,350 (75.4)	1,750 (24.6)	1,539 (85.9)	253 (14.1)
	3	7,296 (72.6)	2,748 (27.4)	5,914 (74.4)	2,038 (25.6)	1,464 (86.5)	229 (13.5)
	4	6,732 (73.1)	2,480 (26.9)	6,936 (73.9)	2,447 (26.1)	1,256 (85.9)	206 (14.1)
	5—most deprived	5,152 (73.8)	1,832 (26.2)	7,259 (74.8)	2,446 (25.2)	956 (84.9)	170 (15.1)
**Sex**	Female	14,574 (72.8)	5,441 (27.2)	13,728 (75.4)	4,473 (24.6)	-	-
	Male	19,580 (73.4)	7,107 (26.6)	16,068 (74.3)	5,561 (25.7)	-	-
**Tumour site**	Colon	19,009 (74.6)	6,488 (25.4)	-	-	-	-
	Rectum	15,145 (71.4)	6,060 (28.6)	-	-	-	-
***31-day target*: *patients who survived at least 90 days*** ^a^							
	**Total**	**112,301 (96.7)**	**3,816 (3.3)**	**84,270 (97.7)**	**1,978 (2.3)**	**15,224 (98.3)**	**271 (1.7)**
**Age groups**	15–44	3,485 (98.4)	57 (1.6)	1,101 (96.8)	36 (3.2)	1,251 (98.3)	21 (1.7)
	45–54	8,131 (98.0)	169 (2.0)	5,206 (97.9)	113 (2.1)	2,302 (98.2)	42 (1.8)
	55–64	22,503 (97.0)	693 (3.0)	17,884 (97.6)	437 (2.4)	3,858 (97.9)	81 (2.1)
	65–74	35,444 (96.5)	1,283 (3.5)	29,759 (97.5)	756 (2.5)	4,528 (98.5)	70 (1.5)
	75+	42,738 (96.4)	1,614 (3.6)	30,320 (97.9)	636 (2.1)	3,285 (98.3)	57 (1.7)
**Stage at diagnosis**	I	13,850 (95.8)	612 (4.2)	14,403 (93.8)	960 (6.2)	3,060 (96.7)	106 (3.3)
	II	22,705 (96.4)	851 (3.6)	8,255 (95.8)	359 (4.2)	904 (97.1)	27 (2.9)
	III	24,168 (96.6)	846 (3.4)	23,309 (98.5)	358 (1.5)	4,344 (98.7)	57 (1.3)
	IV	19,926 (98.4)	322 (1.6)	31,271 (99.5)	171 (0.5)	2,583 (99.4)	16 (0.6)
	Missing	31,652 (96.4)	1,185 (3.6)	7,032 (98.2)	130 (1.8)	4,333 (98.5)	65 (1.5)
**Deprivation quintile**	1—least deprived	23,967 (96.7)	812 (3.3)	11,702 (97.6)	290 (2.4)	3,227 (98.0)	65 (2.0)
	2	24,786 (96.5)	905 (3.5)	14,613 (97.8)	324 (2.2)	3,384 (98.4)	54 (1.6)
	3	23,860 (96.4)	883 (3.6)	16,657 (97.9)	355 (2.1)	3,227 (98.2)	60 (1.8)
	4	22,186 (96.7)	752 (3.3)	19,741 (97.7)	472 (2.3)	3,012 (98.5)	45 (1.5)
	5—most deprived	17,502 (97.4)	464 (2.6)	21,557 (97.6)	537 (2.4)	2,374 (98.1)	47 (1.9)
**Sex**	Female	48,422 (96.8)	1,579 (3.2)	38,729 (97.6)	941 (2.4)	-	-
** **	Male	63,879 (96.6)	2,237 (3.4)	45,541 (97.8)	1,037 (2.2)	-	-
**Tumour site**	Colon	70,337 (96.8)	2,298 (3.2)	-	-	-	-
	Rectum	41,964 (96.5)	1,518 (3.5)	-	-	-	-

^a^ Those with any first course of treatment

The 31-day target, for patients who survived more than 90 days after diagnosis, was more likely to be missed among colorectal cancer patients than lung or ovarian cancer patients (Table 1). Among colorectal cancer patients, the target was more likely to be missed with increasing age, contrasting with a reverse age pattern among those diagnosed with lung cancer. Little difference by age was observed with ovarian cancer. The most deprived patients with a colorectal cancer appeared slightly more likely to be treated within the target time. The proportion of patients for whom the target was missed decreased as stage increased for all three cancer sites (S2–S4 Tables). Supplementary analyses showed that patients undergoing surgical treatment were slightly more likely to have the target missed than those who were first treated with any of the other options (data not shown).

### Exploring the patterns of survival

There was no consistent pattern when survival analyses were stratified by age and stage at diagnosis (Table 2). For patients diagnosed at all stages except stage I, there was evidence that lung cancer patients treated later than 62 days after referral had better survival overall than those managed within the time target (Table 2). Among colorectal cancer patients, differences in one-year survival by target attainment stratified by stage did not show a clear pattern, although there was some evidence that survival was poorer for older patients and patients with late stage for whom the target was met (Table 2).

**Table 2:** One-year net survival for each cancer site, by whether each waiting time target was met or not, England, 2009–13.

			**Colorectal cancer**		**Lung cancer**		**Ovarian cancer**	
**Target attainment**			**met, NS (CI)**	**not met, NS (CI)**	**met, NS (CI)**	**not met, NS (CI)**	**met, NS (CI)**	**not met, NS (CI)**
***TWW pathway***	**Age groups**	15–44	88.9 (86.6–91.2)	90.1 (80.9–99.3)	52.4 (47.7–57.2)	50.1 (14.6–85.5)	92.7 (90.3–95.0)	NA
		45–54	89.0 (87.9–90.1)	89.4 (84.9–93.9)	51.1 (49.2–53.0)	40.7 (29.6–51.8)	91.5 (89.9–93.2)	85.8 (71.2–100.0)
		55–64	86.0 (85.3–86.8)	88.5 (85.5–91.5)	49.0 (48.1–50.0)	53.1 (46.6–59.6)	86.8 (85.3–88.3)	82.0 (69.8–94.2)
		65–74	83.1 (82.4–83.7)	86.5 (83.9–89.2)	46.5 (45.8–47.3)	43.5 (38.8–48.2)	81.6 (80.0–83.2)	79.8 (69.4–90.1)
		75+	76.9 (76.3–77.6)	79.7 (76.9–82.4)	36.2 (35.4–36.9)	37.7 (33.5–41.8)	62.2 (60.0–64.4)	50.2 (36.9–63.6)
	**Stage at diagnosis**	I	99.1 (98.5–99.6)	99.3 (97.1–100.0)	88.8 (87.9–89.7)	84.8 (78.7–91.0)	98.2 (97.3–99.0)	94.1 (86.1–100.0)
		II	95.5 (95.0–96.1)	96.5 (94.1–98.8)	73.5 (72.1–74.9)	76.4 (68.0–84.7)	90.8 (88.1–93.5)	71.2 (58.4–100.0)
		III	90.1 (89.4–90.8)	91.8 (88.9–94.6)	48.1 (47.3–49.0)	46.2 (41.2–51.3)	81.9 (80.3–83.4)	84.9 (74.1–95.6)
		IV	51.7 (50.8–52.7)	54.8 (50.3–59.3)	23.3 (22.8–23.9)	19.5 (16.1–22.9)	65.8 (63.3–68.4)	63.2 (46.5–79.9)
		Missing	80.8 (80.0–81.6)	83.3 (80.1–86.4)	36.7 (35.1–38.4)	37.3 (27.8–46.7)	71.2 (69.3–73.2)	49.3 (35.7–62.9)
**Unstandardised**			81.3 (80.9–81.7)	84.1 (82.5–85.7)	43.5 (43.1–44.0)	42.5 (39.7–45.2)	80.1 (79.3–81.0)	72.4 (65.8–79.1)
***62-day target*** ^a^	**Age groups**	15–44	90.7 (88.3–93.0)	94.0 (90.1–97.9)	55.9 (50.4–61.5)	70.8 (60.5–81.0)	95.2 (93.2–97.2)	NA
		45–54	91.6 (90.5–92.7)	93.9 (92.1–95.8)	57.0 (54.8–59.3)	67.6 (63.4–71.7)	92.9 (91.3–94.5)	91.9 (86.8–97.1)
		55–64	90.3 (89.6–91.1)	91.5 (90.2–92.8)	55.4 (54.2–56.6)	71.2 (69.2–73.2)	90.4 (89.0–91.8)	87.6 (83.4–91.9)
		65–74	89.4 (88.8–90.1)	89.7 (88.7–90.8)	54.2 (53.2–55.1)	69.3 (67.9–70.8)	87.8 (86.3–89.3)	87.8 (84.4–91.3)
		75+	85.4 (84.7–86.1)	90.9 (89.9–91.8)	44.4 (43.4–45.4)	68.3 (66.6–70.1)	76.7 (74.2–79.2)	80.2 (75.6–84.8)
	**Stage at diagnosis**	I	100.0 (NA)	100.0 (NA)	91.2 (90.1–92.3)	93.4 (92.1–94.6)	98.9 (98.1–99.7)	99.3 (97.0–100.0)
		II	98.8 (98.3–99.3)	74.9 (72.3–99.8)	76.4 (74.6–78.2)	81.0 (78.9–83.0)	92.4 (89.7–95.2)	92.4 (85.5–99.4)
		III	93.7 (93.0–94.4)	93.7 (92.6–94.8)	52.4 (51.3–53.4)	65.2 (63.5–67.0)	87.7 (86.1–89.2)	83.6 (79.5–87.6)
		IV	62.8 (61.6–64.0)	71.2 (69.2–73.2)	33.8 (33.0–34.7)	44.6 (42.6–46.7)	76.8 (74.1–79.4)	80.2 (74.4–85.9)
		Missing	88.2 (87.4–89.0)	90.7 (89.5–91.9)	49.3 (47.0–51.6)	61.9 (57.6–66.2)	82.5 (80.6–84.5)	83.3 (78.6–87.9)
**Unstandardised**			88.1 (87.7–88.5)	90.9 (90.2–91.5)	51.3 (50.7–51.8)	69.3 (68.4–70.3)	87.6 (86.7–88.4)	86.2 (84.0–88.4)
***31-day target*** ^a^	**Age groups**	15–44	89.8 (88.8–90.8)	94.8 (89.1–100.0)	60.3 (57.4–63.2)	86.2 (75.1–97.4)	93.4 (92.0–94.8)	NA
		45–54	89.0 (87.9–90.1)	91.0 (90.4–91.6)	57.0 (55.7–58.4)	81.7 (74.6–88.9)	91.8 (90.7–93.0)	93.1 (85.4–100.0)
		55–64	86.0 (85.3–86.8)	91.9 (91.6–92.3)	56.6 (55.9–57.3)	85.6 (82.2–89.0)	88.9 (87.9–89.9)	93.1 (87.4–98.8)
		65–74	83.1 (82.4–83.7)	91.0 (90.7–91.3)	56.7 (56.1–57.2)	86.2 (83.6–88.8)	84.7 (83.6–85.8)	91.1 (84.1–98.2)
		75+	76.9 (76.3–77.6)	85.7 (85.3–86.1)	50.3 (49.7–50.9)	85.2 (82.0–88.5)	73.4 (71.7–75.0)	95.8 (88.1–100.0)
	**Stage at diagnosis**	I	99.8 (99.5–100.0)	100.0 (NA)	89.3 (88.7–89.9)	95.6 (94.0–97.3)	98.5 (97.9–99.1)	98.7 (95.5–100.0)
		II	98.4 (98.1–98.7)	100.0 (NA)	74.4 (73.4–75.4)	86.1 (82.1–90.0)	93.2 (91.4–95.0)	94.3 (84.3–100.0)
		III	93.4 (93.0–93.8)	96.7 (95.0–98.4)	53.9 (53.3–54.6)	74.5 (69.7–79.2)	85.3 (84.2–86.4)	87.2 (78.1–96.2)
		IV	65.2 (64.5–65.9)	81.3 (76.7–85.9)	33.8 (33.2–34.3)	52.6 (45.0–60.2)	74.9 (73.1–76.6)	70.1 (47.8–92.3)
		Missing	89.6 (89.2–90.0)	96.9 (95.5–98.4)	53.0 (51.8–54.2)	83.5 (76.7–90.4)	79.8 (78.6–81.1)	96.5 (91.5–100.0)
**Unstandardised**			89.1 (88.9–89.4)	96.9 (96.1–97.7)	54.4 (54.1–54.8)	85.6 (83.9–87.2)	85.1 (84.5–85.7)	93.7 (90.5–96.9)

^a^ Those with any first course of treatment and who survived at least 90 days after diagnosis

Except for those patients with stage I colorectal cancer, colorectal and lung cancer patients who received their first course of treatment later than 31 days tended to have higher one-year survival than those who were treated shortly after diagnosis (Fig 3 and Table 2). There was an indication of more favourable one-year survival for women with ovarian cancer for whom the target was missed for all stages except stage IV tumours; however, differences were statistically significant only among the group with missing information on stage.

Additional survival analysis by stage and finer waiting time intervals (≤10 days, 11–31 days, 32–45 days, >45 days) revealed that colorectal and lung cancer patients who received treatment within 10 days had lower one-year survival than those who were treated later for all stages (Table 3). This was also true for the unstandardised net survival of ovarian cancer patients, although not when stratified by stage.

**Table 3:** One-year net survival for all patients surviving more than 90 days by waiting time intervals, from decision to treat to first treatment, and by stage, England, 2009–13.

Days (decision to treat to first treatment)		≤10 days	11–31 days	32–45 days	>45 days
		NS% (95% CI)	NS% (95% CI)	NS% (95% CI)	NS% (95% CI)
**Colorectal cancer**					
**Stage**	I	99.1 (98.6–99.6)	100.0 (NA)	100.0 (NA)	98.9 (96.2–100.0)
	II	96.8 (96.2–97.3)	99.8 (99.5–100.0)	100.0 (NA)	98.7 (96.4–100.0)
	III	90.2 (89.5–90.8)	96.0 (95.5–96.4)	97.0 (95.1–99.0)	96.1 (92.8–99.4)
	IV	59.0 (58.1–59.9)	74.7 (73.7–75.7)	81.0 (75.7–86.2)	82.1 (72.7–91.6)
	Missing	85.5 (84.9–86.1)	94.5 (94.0–95.0)	97.3 (95.6–99.1)	96.0 (93.3–98.7)
Unstandardised		84.2 (83.9–84.6)	94.1 (93.8–94.3)	97.0 (96.0–97.9)	96.7 (95.2–98.2)
**Lung cancer**					
**Stage**	I	84.8 (83.8–85.8)	93.0 (92.4–93.7)	94.3 (92.0–96.6)	98.0 (95.8–100.0)
	II	68.8 (67.2–70.3)	79.7 (78.4–80.9)	84.8 (79.9–89.7)	88.8 (82.4–95.3)
	III	49.2 (48.3–50.0)	60.1 (59.1–61.1)	73.1 (67.7–78.6)	79.2 (69.7–88.6)
	IV	30.9 (30.3–31.6)	39.9 (38.9–40.9)	49.6 (41.2–57.9)	67.4 (50.0–84.7)
	Missing	48.4 (46.9–49.8)	62.9 (60.8–64.9)	84.0 (76.0–92.0)	81.9 (69.0–94.8)
Unstandardised		47.5 (47.1–48.0)	64.5 (63.9–65.0)	83.1 (81.0–85.2)	91.4 (88.8–93.9)
**Ovarian cancer**					
**Stage**	I	97.9 (96.9–98.8)	99.0 (98.2–99.7)	99.0 (95.5–100.0)	97.7 (90.7–100.0)
	II	92.3 (89.4–95.2)	93.9 (91.6–96.2)	90.6 (76.1–100.0)	NA
	III	83.7 (82.0–85.3)	87.0 (85.5–88.5)	83.9 (72.4–95.4)	94.6 (83.1–100.0)
	IV	72.9 (70.5–75.3)	77.2 (74.7–79.6)	67.7 (42.1–93.3)	76.5 (38.7–100.0)
	Missing	76.0 (74.3–77.8)	92.9 (89.0–96.8)	97.2 (91.7–100.0)	94.5 (83.0–100.0)
Unstandardised		82.3 (81.4–83.2)	88.0 (87.2–88.8)	92.9 (89.0–96.8)	95.9 (90.5–100.0)

### Time-to-treatment analyses

The number and proportion of patients receiving each type of treatment, for each target, is shown in Table 4. The univariate analysis shows the pattern of unadjusted time to treatment i.e. the days actually experienced by the patients. For patients diagnosed through urgent GP referral and who survived more than 90 days after diagnosis, half of those with colorectal, non-small cell lung or ovarian cancer had had their treatment by 55, 53 and 49 days after referral, respectively. After the decision to treat was made, half of the patients were treated by 11, 7 and 10 days respectively (S2 Fig).

**Table 4:** Type of first treatment received by patients (any referral pathway) who survived at least 90 days after diagnosis, for each cancer site by stage.

	Stage I	Stage II	Stage III	Stage IV	Missing	Total
	n (%)	n (%)	n (%)	n (%)	n (%)	n (%)
**Colorectal cancer**						
surgery	11,679 (81.0)	19,697 (83.7)	18,814 (75.3)	10,731 (53.1)	24,723 (75.6)	85,644 (73.9)
anti-cancer drug regimen	186 (1.3)	470 (2.0)	825 (3.3)	5,342 (26.4)	1,530 (4.7)	8,353 (7.2)
radiotherapy	2,085 (14.5)	2,703 (11.5)	4,470 (17.9)	1,762 (8.7)	3,577 (10.9)	14,597 (12.6)
palliative	139 (1.0)	242 (1.0)	415 (1.7)	1,644 (8.1)	1,471 (4.5)	3,911 (3.4)
active monitoring	270 (1.9)	394 (1.7)	405 (1.6)	673 (3.3)	1,308 (4.0)	3,050 (2.6)
treatment declined	57 (0.4)	29 (0.1)	54 (0.2)	53 (0.3)	110 (0.3)	303 (0.3)
**Total**	**14,416 (100.0)**	**23,535 (100.0)**	**24,983 (100.0)**	**20,205 (100.0)**	**32,719 (100.0)**	**115,858 (100.0)**
**Lung cancer**						
surgery	9,343 (61.1)	4,372 (50.9)	2,735 (11.6)	1,602 (5.1)	1,435 (20.2)	19,487 (22.7)
anti-cancer drug regimen	462 (3.0)	987 (11.5)	10,102 (42.9)	15,969 (51.1)	2,421 (34.0)	29,941 (34.9)
radiotherapy	2,781 (18.2)	1,746 (20.3)	6,179 (26.2)	6,289 (20.1)	1,285 (18.1)	18,280 (21.3)
palliative	909 (5.9)	625 (7.3)	2,310 (9.8)	4,296 (13.7)	1,025 (14.4)	9,165 (10.7)
active monitoring	1,749 (11.4)	834 (9.7)	2,180 (9.2)	2,990 (9.6)	917 (12.9)	8,670 (10.1)
treatment declined	37 (0.2)	30 (0.3)	69 (0.3)	134 (0.4)	32 (0.4)	302 (0.4)
**Total**	**15,281 (100.0)**	**8,594 (100.0)**	**23,575 (100.0)**	**31,280 (100.0)**	**7,115 (100.0)**	**85,845 (100.0)**
**Ovarian cancer**						
surgery	3,086 (97.5)	857 (92.1)	2,169 (49.3)	621 (23.9)	1,997 (45.5)	8,730 (56.4)
anti-cancer drug regimen	64 (2.0)	67 (7.2)	2,139 (48.6)	1,849 (71.2)	2,090 (47.6)	6,209 (40.1)
radiotherapy	0 (0.0)	3 (0.3)	27 (0.6)	25 (1.0)	61 (1.4)	116 (0.7)
palliative	6 (0.2)	4 (0.4)	46 (1.0)	81 (3.1)	162 (3.7)	299 (1.9)
active monitoring	9 (0.3)	0 (0.0)	17 (0.4)	19 (0.7)	74 (1.7)	119 (0.8)
treatment declined	1 (0.0)	0 (0.0)	3 (0.1)	1 (0.0)	9 (0.2)	14 (0.1)
**Total**	**3,166 (100.0)**	**931 (100.0)**	**4,401 (100.0)**	**2,596 (100.0)**	**4,393 (100.0)**	**15,487 (100.0)**

Footnote: excludes those given ‘other therapies

As expected, treatment was rarely given straight after referral (62-day target): the proportion started to rise from around 10–15 days, once patients were first seen. There was a steep increase in the rate of treatment given as the 62-day target approached and a clear deceleration of treatments given after that ‘cut-off’. After the decision to treat was made, a proportion of patients were given treatment very early in time for all these cancers (around 20–30% within 2–3 days), with a slow, steady increase in the proportion treated from 3 to 31 days and a distinct plateau at 31 days (S2 Fig).

**Fig 4:**
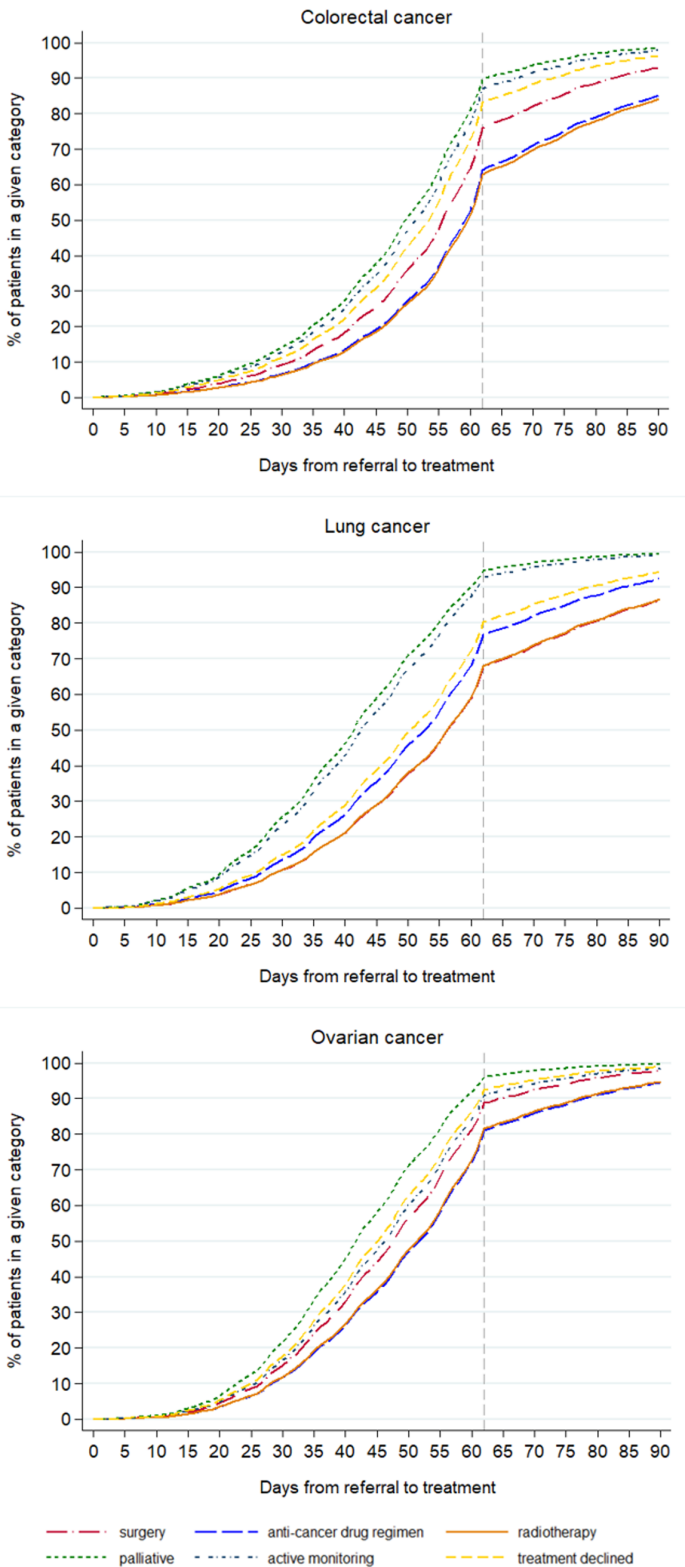
Time to first treatment (62-day target) by treatment category for cancer patients who survived 90 days after diagnosis, England, 2009–2013.

**Fig 5:**
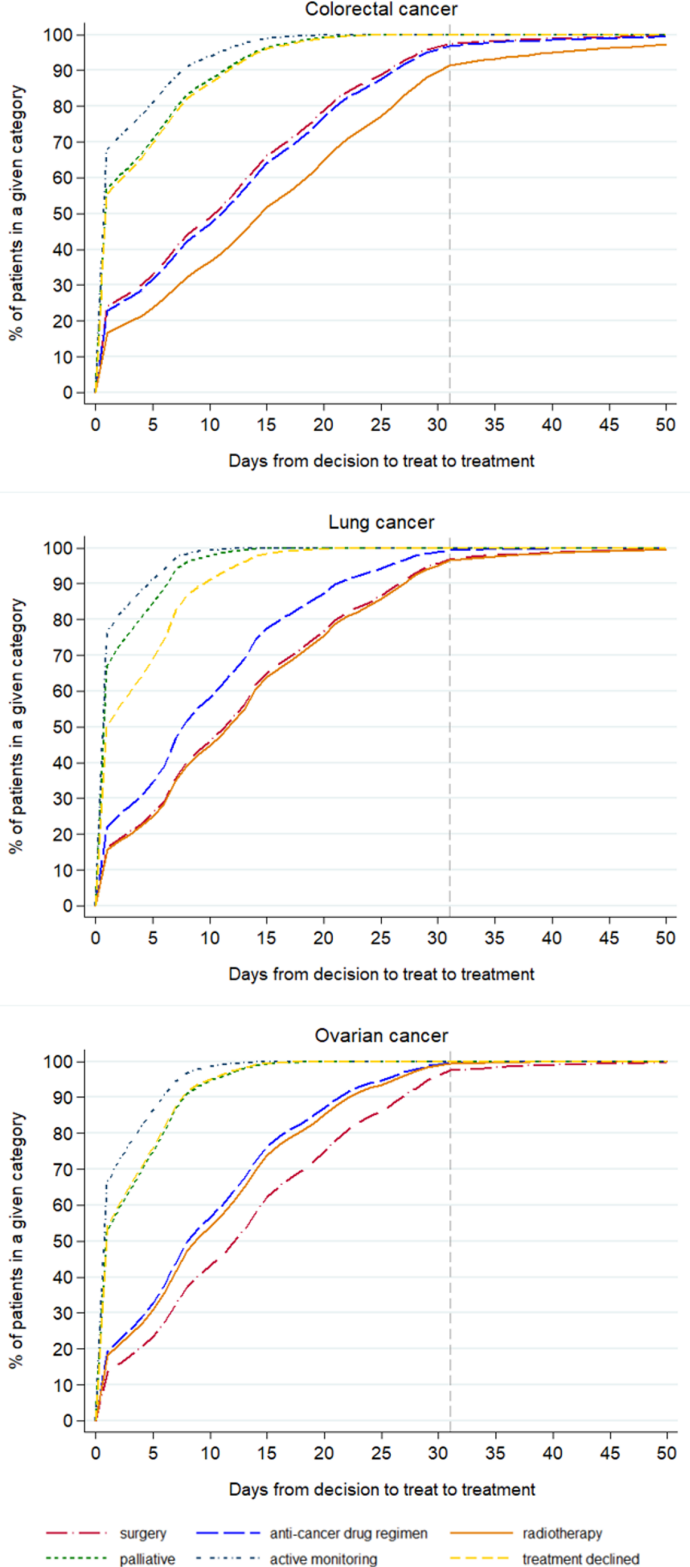
Time to first treatment (31-day target) by treatment category for cancer patients who survived 90 days after diagnosis, England, 2009–2013.

The pattern of the timing of the different treatments given, after adjusting for age, stage and deprivation is shown in Figs 4 and 5. For all the cancers, the earliest treatments to be recorded, counting from either referral or from decision to treatment, were palliative care, active monitoring and when treatment was declined. The treatments that started later were surgery, radiotherapy and an anti-cancer drug regimen, the order changing depending on the cancer site (Figs 4 and 5).

## Discussion

### Summary of main findings

The great majority of patients diagnosed with colorectal, lung and ovarian cancers had the TWW and the 31-day targets met, but the 62-day target was met less often, especially with the increasing age of the patients. One-year net survival was higher in patients for whom the 31- and 62-day targets were not met than for those whom the target was met. Lower survival among patients treated more quickly was mainly driven by the lower survival of those who received a first course of treatment shortly after diagnosis (≤10 days). The time-to-treatment analyses showed that palliative care and active monitoring were consistently given to patients earliest in time, even after adjusting for age, stage and deprivation. Patients’ refusal of treatment was also recorded soon after diagnosis. Therefore, the somewhat counter-intuitive result of lower survival for those for whom the targets were met, may be explained by the early receipt of palliative treatments by those patients who were likely in any case to have had the poorer prognosis, while treatments which were potentially of curative intent were more likely to have been given after a longer period of time.

### Strengths and weaknesses of the study

We used the dates recorded in CWT for official monitoring of the targets, so we can be confident in the correspondence of these results with official statistics reporting waiting times performance. Our analyses included a large set of individually-linked records combining information from several sources, enabling us to describe the characteristics of the patients and stratify the analyses where appropriate. There were some limitations to the data that have constrained the analyses we could undertake: there was not the possibility, for example, to stratify by exact type of treatment given. However, neither is this done for the official monitoring of the CWT targets.

Around one fifth of colorectal, lung and ovarian cancer patients in the cancer registry data were not included in this analysis, as they did not have a matching record in CWT. Some of these patients did not appear in CWT because they received treatment in private settings (the King’s Fund reports that about “11 per cent of the UK population has some form of private medical insurance” (p3, [38]). Others will have died before treatment could commence: in our data, of those without a CWT record, 22%, 44% and 31% of colorectal, lung and ovarian cancer patients, respectively, died within 30 days of diagnosis. However, a significant proportion survived at least 30 days, which would have allowed time to plan and start treatment, especially palliative care. There is a possibility, therefore, that the associations between waiting time and survival are different for these ‘missing’ patients than the results of this study [39]. These potential sources of bias add to the argument that caution should be used when interpreting CWT data available to the policy-makers for the evaluation of cancer outcomes.

We have focused in this study on one-year net survival as we can use the most up-to-date data available to us and that is often used for monitoring purposes. It also highlights a short-term outcome for patients during a period in which the effect of treatment is most relevant. As we are most interested in the time covered by the monitoring framework, and unless we have specific hypotheses about certain treatments, looking at longer-term outcomes is unlikely to give additional information about the utility of meeting targets. We know too, that there are possible limitations in the accuracy of the categorisation of the treatment variables in CWT. For example, the time-to-treatment graphs show the category of ‘active monitoring’ being given anywhere up to 15–20 days after a decision to treat is recorded, whereas we would expect active monitoring to start on the same day as the decision to treat. Variations in the interpretation of this category by clinical staff might explain why the time to this ‘treatment’ is not always zero. Those recorded as receiving palliative treatments after being diagnosed at stage I or II tend to be the oldest patients. However, there are cases of younger patients, some with no comorbidities recorded, also having a record of palliative treatment at an early stage of disease, which suggests some inaccurate categorisation or recording. Nonetheless, the stage-specific time-to-treatment graphs (S3–S5 Figs) show that those with stage IV disease receive all types of treatment earlier than those with stage I or II, which helps to explain the seemingly paradoxical finding of worse survival for those treated within targets. Similarly, the current categorisation of the CWT treatment variable does not allow for fully distinguishing within the “surgery” category between curative and palliative intent, or for assessing the appropriateness of treatment with regard to the disease stage. These limitations in the nature of the data do not affect the survival estimates found, but they do mean that it is not possible to separate those patients for whom the times between referral, decision to treat and start of treatment could actually have an impact on the clinical outcomes.

This is the only study we are aware of that has used individually-linked population-based sources of data to examine the association with survival of the official English National Cancer Waiting Time targets. Other studies have examined waiting times from diagnosis to specific treatments and outcomes from selected cancers, with mixed findings, some pointing to an association between higher mortality and longer times to treatment [13, 14] or to a lack of influence on long-term outcomes [26, 27, 30, 40]. Several have examined the impact of the introduction of the TWW on outcomes for specific cancer sites, but, again, the evidence is mixed with regards to the benefits [2, 4, 28, 41].

Several studies in other countries, and in some without relation to specific waiting time targets, have examined the relationship between the time patients wait between referral or diagnosis and treatment, and their outcomes. Some of these have highlighted the same “waiting time paradox” [3] that we have found, in which people with worse symptoms are more likely to be seen and treated more quickly, but have poorer outcomes as they are more likely to be diagnosed at a later stage [3, 5, 42, 43]. We found this to be the case even though we had excluded patients who died within 90 days. If we had included these patients, the effect of the “waiting time paradox” would have been even more pronounced, as is seen in S1 Fig. This phenomenon, also named confounding by indication, may result from the shortest delay being experienced by patients with more advanced diseases or concurrent chronic conditions [42, 44, 45]. In this case, it is likely that the “sicker quicker” effect [44] plays a role and that the sickest patients (e.g. those presenting with a bowel occlusion in the case of a colorectal cancer) gain access to palliative and symptom-relief treatments quickly, although these may not have any positive impact on survival. In contrast, major surgery, chemotherapy or radiotherapy regimes, which are more likely to be performed with curative intent and have a positive impact on survival, may require more time in which to plan their implementation: for instance, patients may first need to undergo further diagnostic investigations which lengthen the interval between diagnosis and start of treatment. At the same time, they are more resource intensive and therefore need to be scheduled in accordance with the available human and logistic assets (e.g. number of anaesthetists and bed availability), which are likely to be constrained, especially in a time of health care spending cuts [46]. These considerations mean that patients who are more likely to benefit from the treatment are those who are more likely to receive it somewhat later, as confirmed by our analyses. Where any delays result from limited resources rather than clinical need, this could be a cause for concern as these delays may make the option of curative treatment ultimately less likely.

The varied evidence for the association of waiting times with short- and longer-term outcomes implies that the cut off times of these targets are somewhat arbitrary, with 31 and 62 days having little biological relevance: indeed, some studies point out the difficulty of showing a difference in outcomes that depends on short time differences [26, 30]. However, there is general agreement that intervals between referral, diagnosis and treatment should be minimised, as every effort should be made to reduce the anxiety experienced by patients while waiting, however long that is [8–10, 24, 29]. Murchie et al point out that the publicity surrounding waiting times could itself engender anxiety when patients have to wait past the ‘cut-off’ [27], and this anxiety can have wider impacts: a recent study reported the highest mortality rates among the most distressed cancer patients [12].

### Potential future research

This study demonstrated that it is not the times between referral, decision to treat and start of treatment alone that matter for patients’ outcomes. Accounting for the type of treatment would therefore be useful for assessing whether being treated within a certain time leads to better outcomes. Having reliable information on treatment and on its intent (i.e. curative or palliative) would help to better define meaningful groups of patients and understand more fully for which group shorter times would make a difference in survival. However, this would mean to access other relevant datasets and not relying solely on the official data in CWT. Furthermore, the operational standards for these targets are assessed for national statistics with all cancers combined. However, the results found by this study varied by cancer, an insight which was possible thanks to the analysis of individual-level data. Further analysis that takes into account sub-types of cancer, where the data allow, would also be informative as these often call for very different treatment regimens.

Equally important would be to establish whether longer intervals between diagnosis and the start of treatment do decrease the probability of curative treatment being given; and to understand whether longer delays before treatment of curative intent is provided, which may allow for better planning and preparation, improves outcomes. These could add to our understanding of the efficacy of these time targets, and lead to higher cancer survival.

The recently-proposed new target, to ensure that patients with a suspicion of cancer are told within 28 days from GP referral whether they have a cancer or not, will need to be evaluated. CWT is being updated to collect additional information which will enable this to be monitored in future, including a date when the patient is informed of a diagnosis or the ruling out of cancer. As important as this may be for patients’ experience, it is yet to be shown, however, that there will be any difference in patients’ survival in relation to this new target. Continuing focus should also be on whether being diagnosed through a certain pathway (e.g. TWW) makes a difference compared to other pathways (e.g. non-urgent GP referral).

### Implications

Cancer Waiting Time targets may still be important as indicators of overall performance of a health service, and having these targets has set cancer apart from other diseases, enabling cancer patients to be treated more quickly. This is positive as extensive delays would undoubtedly have clinical consequences on outcomes. Waiting time targets can also act as empowering tools for both patients and health professionals who can use them to push for timely decisions and efficient use of resources. As such, they encourage the system to be more equitable and ensure that the majority of patients are treated within the set time frame, reducing stress and anxiety. The targets have been updated since their first introduction to maintain this focus on improving services. However, based on these individually-linked data, and for the cancers we looked at, it is important for patients and policy-makers to appreciate that Cancer Waiting Time targets being met does not necessarily translate into improved one-year survival, due the clinical conditions of the patients at diagnosis and the fact that curative treatments may take more time to plan and implement.

The CWT targets, reported for all cancers together despite a variety of different curative treatments, are not necessarily informative, and do not inevitably lead to improved clinical outcomes. The composition of the data, which currently makes no distinction between surgery with curative and palliative intent, and therefore limited our ability to draw conclusions beyond those presented here, also means that the targets are less informative with regards to the assessment of outcomes. Therefore, survival cannot be used to judge the utility of these targets, nor can the waiting time target-meeting performance of Trusts indicate how good the survival of their patients is likely to be. The other positive aspects of having reduced waiting times for patients and improved overall efficiency of the system, should be seen as more relevant than improved survival.

## Supporting information

S1 Fig Net survival curves for cancer patients, from diagnosis to one year, by target attainment, England, 2009–13. (DOCX)

S2 Fig Time to treatment (univariate analysis) for cancer patients who survived 90 days after diagnosis, England, 2009–13. (DOCX)

S3 Fig Stage-specific time-to-treatment graphs for colorectal cancer patients (stage I/II and stage IV for each target). (DOCX)

S4 Fig Stage-specific time-to-treatment graphs for lung cancer patients (stage I/II and stage IV for each target). (DOCX)

S5 Fig Stage-specific time-to-treatment graphs for ovarian cancer patients (stage I/II and stage IV for each target). (DOCX)

S1 Table Treatment categories in the analysis and list of treatments they cover, as defined in the Cancer Waiting Time monitoring dataset. (DOCX)

S2 Table TWW target attainment by patient characteristics for each cancer site, by stage, England, 2009–13. (DOCX)

S3 Table 62-day target attainment by patient characteristics for each cancer site, by stage, England, 2009–13. (DOCX)

S4 Table 31-day target attainment by patient characteristics for each cancer site, by stage, England, 2009–13. (DOCX)
