# Supplementary material for: Can we assess Cancer Waiting Time targets with cancer survival? A population-based study of individually linked data from the National Cancer Waiting Times monitoring dataset in England, 2009-2013
Source: PLoS One. 2018 Aug 22;13(8):e0201288. doi: 10.1371/journal.pone.0201288 (PMC6104918; doi:10.1371/journal.pone.0201288)
Supplement: S4 Fig — (DOCX) [file pone.0201288.s004.docx]

62-day target

31-day target


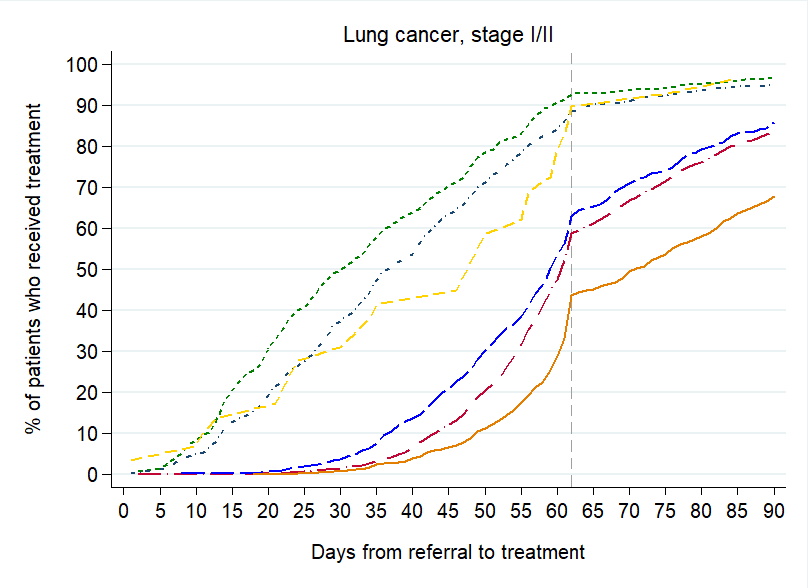

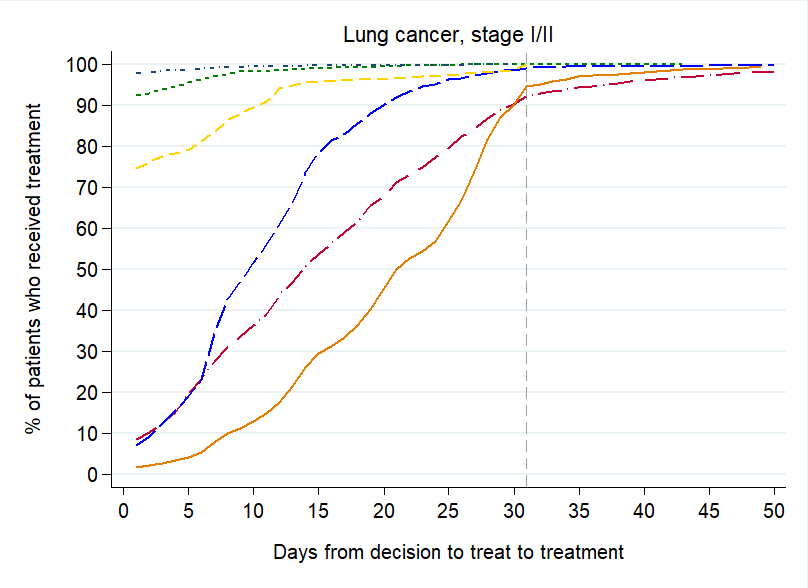

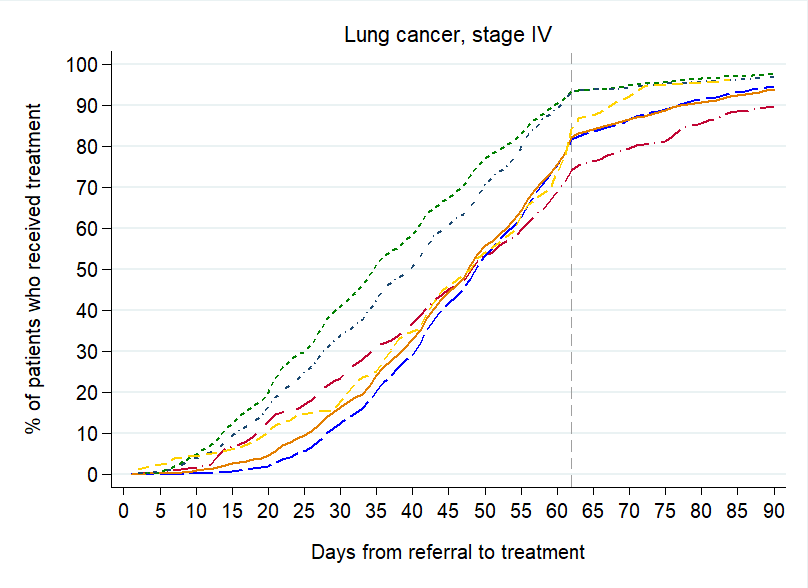

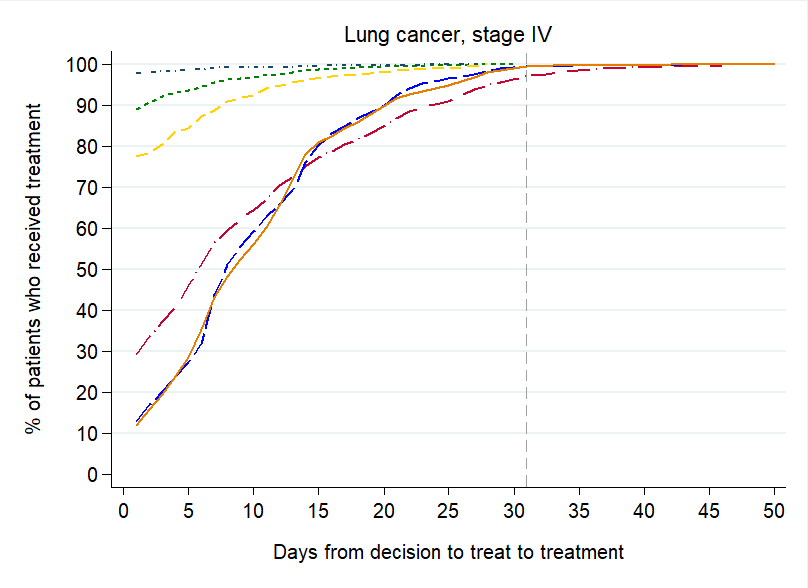


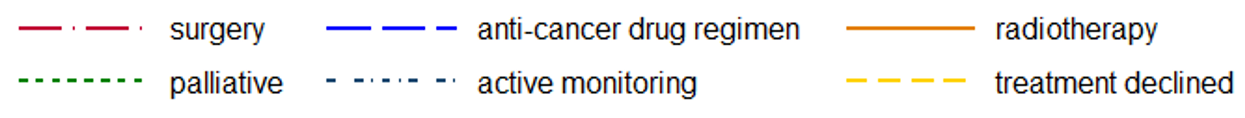


**S4 Fig: Stage-specific time-to-treatment graphs for lung cancer patients (stage I/II and stage IV for each target)**
