# Supplementary material for: Can we assess Cancer Waiting Time targets with cancer survival? A population-based study of individually linked data from the National Cancer Waiting Times monitoring dataset in England, 2009-2013
Source: PLoS One. 2018 Aug 22;13(8):e0201288. doi: 10.1371/journal.pone.0201288 (PMC6104918; doi:10.1371/journal.pone.0201288)
Supplement: S1 Table — (DOCX) [file pone.0201288.s006.docx]

**S1 Table: Treatment categories in the analysis and list of treatments they cover, as defined in the Cancer Waiting Time monitoring dataset**

| **Treatment category** | **Treatment in the CWT dataset [modality code as reported in CWT]** |
| --- | --- |
| **Surgery** | Surgery, radiosurgery [1, 22] |
| **Anti-cancer drug regimen** | Anti-cancer drug regimen (cytotoxic chemotherapy), anti-cancer drug regimen (hormone therapy), anti-cancer drug regimen (other), anti-cancer drug regimen (immunotherapy) [2, 3, 14, 15] |
| **Radiotherapy** | Chemoradiotherapy, teletherapy (beam radiation excluding proton therapy), brachytherapy, proton therapy, radioisotope therapy (including radioiodine) [4, 5, 6, 13, 19] |
| **Palliative** | Specialist palliative care, non-specialist palliative care (excluding active monitoring) [7, 9] |
| **Active monitoring** | Active monitoring (excluding non-specialist palliative care) [8] |
| **Treatment declined** | All treatment declined by the patient [97, 98] |
| **Other therapies (excluded)** | Radio frequency ablation, high intensity focussed ultrasound, cryotherapy, light therapy (including Photodynamic Therapy and Psoralen and Ultraviolet A Therapy – PUVA), hyperbaric oxygen therapy, laser treatment (including Argon beam therapy), biological therapies (excluding immunotherapy), other treatment [10, 11, 12, 16, 18, 20, 21, 23] |
