# Supplementary material for: Can we assess Cancer Waiting Time targets with cancer survival? A population-based study of individually linked data from the National Cancer Waiting Times monitoring dataset in England, 2009-2013
Source: PLoS One. 2018 Aug 22;13(8):e0201288. doi: 10.1371/journal.pone.0201288 (PMC6104918; doi:10.1371/journal.pone.0201288)
Supplement: S2 Table — (DOCX) [file pone.0201288.s007.docx]

**S2 Table: TWW target attainment by patient characteristics for each cancer site, by stage, England, 2009-13**

| **Colorectal cancer** | | | **Stage I** | | | **Stage II** | | **Stage III** | | **Stage IV** | | | **Missing** | | | **All stages** | |  |
| --- | --- | --- | --- | --- | --- | --- | --- | --- | --- | --- | --- | --- | --- | --- | --- | --- | --- | --- |
| **TWW Target attainment** | | | **met**  **n (%)** | **not met**  **n (%)** | | **met**  **n (%)** | **not met**  **n (%)** | **met**  **n (%)** | **not met**  **n (%)** | **met**  **n (%)** | **not met**  **n (%)** | | **met**  **n (%)** | **not met**  **n (%)** | | **met**  **n (%)** | **not met**  **n (%)** |  |
| **Age groups** | | |  |  | |  |  |  |  |  |  | |  |  | |  |  |  |
| 15-44 | | | 68 (91.9) | 6 (8.1) | | 123 (98.4) | 2 (1.6) | 184 (92.9) | 14 (7.1) | 178 (95.7) | 8 (4.3) | | 168 (94.4) | 10 (5.6) | | **721 (94.7)** | **40 (5.3)** |  |
| 45-54 | | | 358 (95.0) | 19 (5.0) | | 525 (93.4) | 37 (6.6) | 788 (93.8) | 52 (6.2) | 823 (94.8) | 45 (5.2) | | 674 (95.6) | 31 (4.4) | | **3,168 (94.5)** | **184 (5.5)** |  |
| 55-64 | | | 919 (93.5) | 64 (6.5) | | 1,473 (95.0) | 77 (5.0) | 1,979 (94.9) | 106 (5.1) | 2,165 (95.4) | 104 (4.6) | | 2,048 (94.8) | 113 (5.2) | | **8,584 (94.9)** | **464 (5.1)** |  |
| 65-74 | | | 1,496 (93.7) | 100 (6.3) | | 2,679 (94.6) | 154 (5.4) | 3,073 (95.5) | 144 (4.5) | 3,143 (96.2) | 125 (3.8) | | 3,452 (94.8) | 191 (5.2) | | **13,843 (95.1)** | **714 (4.9)** |  |
| 75+ | | | 2,237 (95.1) | 116 (4.9) | | 4,944 (95.0) | 259 (5.0) | 4,399 (95.8) | 193 (4.2) | 4,368 (95.3) | 215 (4.7) | | 6,177 (94.9) | 329 (5.1) | | **22,125 (95.2)** | **1,112 (4.8)** |  |
| **Deprivation quintile** | | | | | |  |  |  |  |  |  | |  |  | |  |  |  |
| 1 - least deprived | | | 1,115 (95.5) | 53 (4.5) | | 2,132 (95.1) | 109 (4.9) | 2,207 (95.9) | 94 (4.1) | 2,257 (96.4) | 85 (3.6) | | 2,577 (94.3) | 156 (5.7) | | **10,288 (95.4)** | **497 (4.6)** |  |
| 2 | | | 1,191 (94.0) | 76 (6.0) | | 2,206 (94.5) | 128 (5.5) | 2,330 (96.2) | 93 (3.8) | 2,376 (95.7) | 108 (4.3) | | 2,732 (95.0) | 144 (5.0) | | **10,835 (95.2)** | **549 (4.8)** |  |
| 3 | | | 1,044 (94.9) | 56 (5.1) | | 2,075 (94.7) | 116 (5.3) | 2,239 (95.1) | 115 (4.9) | 2,287 (94.9) | 123 (5.1) | | 2,792 (95.5) | 132 (4.5) | | **10,437 (95.1)** | **542 (4.9)** |  |
| 4 | | | 996 (94.2) | 61 (5.8) | | 1,867 (95.2) | 95 (4.8) | 2,054 (95.6) | 95 (4.4) | 2,167 (95.3) | 106 (4.7) | | 2,567 (95.2) | 130 (4.8) | | **9,651 (95.2)** | **487 (4.8)** |  |
| 5 - most deprived | | | 732 (92.5) | 59 (7.5) | | 1,464 (94.8) | 81 (5.2) | 1,593 (93.4) | 112 (6.6) | 1,590 (95.5) | 75 (4.5) | | 1,851 (94.3) | 112 (5.7) | | **7,230 (94.3)** | **439 (5.7)** |  |
| **Sex** | | |  |  | |  |  |  |  |  |  | |  |  | |  |  |  |
| Female | | | 2,113 (94.0) | 136 (6.0) | | 4,291 (94.7) | 240 (5.3) | 4,365 (95.3) | 217 (4.7) | 4,348 (95.6) | 198 (4.4) | | 5,625 (94.7) | 315 (5.3) | | **20,742 (94.9)** | **1,106 (5.1)** |  |
| Male | | | 2,965 (94.6) | 169 (5.4) | | 5,453 (95.0) | 289 (5.0) | 6,058 (95.4) | 292 (4.6) | 6,329 (95.5) | 299 (4.5) | | 6,894 (95.1) | 359 (4.9) | | **27,699 (95.2)** | **1,408 (4.8)** |  |
| **Tumour site** | | |  |  | |  |  |  |  |  |  | |  |  | |  |  |  |
| colon | | | 1,954 (93.3) | 141 (6.7) | | 6,119 (94.9) | 331 (5.1) | 5,242 (95.5) | 248 (4.5) | 6,376 (96.1) | 259 (3.9) | | 7,347 (94.7) | 413 (5.3) | | **27,038 (95.1)** | **1,392 (4.9)** |  |
| rectum | | | 3,124 (95.0) | 164 (5.0) | | 3,625 (94.8) | 198 (5.2) | 5,181 (95.2) | 261 (4.8) | 4,301 (94.8) | 238 (5.2) | | 5,172 (95.2) | 261 (4.8) | | **21,403 (95.0)** | **1,122 (5.0)** |  |
| **Total** | | | **5,078 (94.3)** | **305 (5.7)** | | **9,744 (94.9)** | **529 (5.1)** | **10,423 (95.3)** | **509 (4.7)** | **10,677 (95.6)** | **497 (4.4)** | | **12,519 (94.9)** | **674 (5.1)** | | **48,441 (95.1)** | **2,514 (4.9)** |  |
| **Lung cancer** | | **Stage I** | | | | **Stage II** | | **Stage III** | | **Stage IV** | | **Missing** | | | **All stages** | | | |
| **TWW Target attainment** | | **met**  **n (%)** | | **not met**  **n (%)** | | **met**  **n (%)** | **not met**  **n (%)** | **met**  **n (%)** | **not met**  **n (%)** | **met**  **n (%)** | **not met**  **n (%)** | **met**  **n (%)** | | **not met**  **n (%)** | **met**  **n (%)** | | **not met**  **n (%)** | |
| **Age groups** | |  | |  | |  |  |  |  |  |  |  | |  |  | |  | |
|  | 15-44 | 44 (100.0) | | 0 (0.0) | | 31 (96.9) | 1 (3.1) | 99 (97.1) | 3 (2.9) | 217 (99.5) | 1 (0.5) | 33 (97.1) | | 1 (2.9) | **424 (98.6)** | | **6 (1.4)** | |
|  | 45-54 | 258 (96.6) | | 9 (3.4) | | 198 (98.0) | 4 (2.0) | 784 (97.6) | 19 (2.4) | 1,277 (97.6) | 31 (2.4) | 186 (94.4) | | 11 (5.6) | **2,703 (97.3)** | | **74 (2.7)** | |
|  | 55-64 | 1,063 (97.3) | | 29 (2.7) | | 898 (98.2) | 16 (1.8) | 3,061 (97.7) | 71 (2.3) | 4,663 (97.9) | 100 (2.1) | 690 (98.3) | | 12 (1.7) | **10,375 (97.8)** | | **228 (2.2)** | |
|  | 65-74 | 2,255 (97.7) | | 54 (2.3) | | 1,619 (97.5) | 41 (2.5) | 5,172 (97.5) | 130 (2.5) | 7,786 (97.7) | 183 (2.3) | 1,073 (96.8) | | 35 (3.2) | **17,905 (97.6)** | | **443 (2.4)** | |
|  | 75+ | 2,363 (96.6) | | 83 (3.4) | | 1,598 (96.7) | 54 (3.3) | 4,934 (96.5) | 180 (3.5) | 7,980 (97.3) | 222 (2.7) | 1,498 (97.0) | | 47 (3.0) | **18,373 (96.9)** | | **586 (3.1)** | |
| **Lung cancer** | | **Stage I** | | | | **Stage II** | | **Stage III** | | **Stage IV** | | **Missing** | | | **All stages** | | | |
| **TWW Target attainment** | | **met**  **n (%)** | | **not met**  **n (%)** | | **met**  **n (%)** | **not met**  **n (%)** | **met**  **n (%)** | **not met**  **n (%)** | **met**  **n (%)** | **not met**  **n (%)** | **met**  **n (%)** | | **not met**  **n (%)** | **met**  **n (%)** | | **not met**  **n (%)** | |
| **Deprivation quintile** | | | |  | |  |  |  |  |  |  |  | |  |  | |  | |
| 1 - least deprived | | 799 (97.9) | | 17 (2.1) | | 604 (97.1) | 18 (2.9) | 1,868 (98.1) | 37 (1.9) | 3,273 (98.2) | 59 (1.8) | 520 (97.2) | | 15 (2.8) | **7,064 (98.0)** | | **146 (2.0)** | |
|  | 2 | 1,020 (96.8) | | 34 (3.2) | | 762 (98.6) | 11 (1.4) | 2,416 (97.0) | 74 (3.0) | 3,957 (98.0) | 82 (2.0) | 648 (97.3) | | 18 (2.7) | **8,803 (97.6)** | | **219 (2.4)** | |
|  | 3 | 1,160 (97.3) | | 32 (2.7) | | 858 (97.1) | 26 (2.9) | 2,819 (97.2) | 82 (2.8) | 4,518 (97.6) | 109 (2.4) | 693 (96.8) | | 23 (3.2) | **10,048 (97.4)** | | **272 (2.6)** | |
|  | 4 | 1,433 (96.9) | | 46 (3.1) | | 1,020 (97.6) | 25 (2.4) | 3,412 (96.8) | 112 (3.2) | 5,120 (97.5) | 131 (2.5) | 844 (96.9) | | 27 (3.1) | **11,829 (97.2)** | | **341 (2.8)** | |
| 5 - most deprived | | 1,571 (97.2) | | 46 (2.8) | | 1,100 (96.8) | 36 (3.2) | 3,535 (97.3) | 98 (2.7) | 5,055 (97.0) | 156 (3.0) | 775 (97.1) | | 23 (2.9) | **12,036 (97.1)** | | **359 (2.9)** | |
| **Sex** | |  | |  | |  |  |  |  |  |  |  | |  |  | |  | |
|  | Female | 3,051 (97.0) | | 93 (3.0) | | 1,839 (97.6) | 46 (2.4) | 6,133 (97.2) | 174 (2.8) | 9,645 (97.5) | 250 (2.5) | 1,629 (97.0) | | 50 (3.0) | **22,297 (97.3)** | | **613 (2.7)** | |
|  | Male | 2,932 (97.3) | | 82 (2.7) | | 2,505 (97.3) | 70 (2.7) | 7,917 (97.2) | 229 (2.8) | 12,278 (97.7) | 287 (2.3) | 1,851 (97.1) | | 56 (2.9) | **27,483 (97.4)** | | **724 (2.6)** | |
| **Total** | | **5,983 (97.2)** | | **175 (2.8)** | | **4,344 (97.4)** | **116 (2.6)** | **14,050 (97.2)** | **403 (2.8)** | **21,923 (97.6)** | **537 (2.4)** | **3,480 (97.0)** | | **106 (3.0)** | **49,780 (97.4)** | | **1,337 (2.6)** | |
| **Ovarian cancer** | | **Stage I** | | | | **Stage II** | | **Stage III** | | **Stage IV** | | **Missing** | | | **All stages** | | | |
| **TWW Target attainment** | | **met**  **n (%)** | | | **not met**  **n (%)** | **met**  **n (%)** | **not met**  **n (%)** | **met**  **n (%)** | **not met**  **n (%)** | **met**  **n (%)** | **not met**  **n (%)** | **met**  **n (%)** | | **not met**  **n (%)** | **met**  **n (%)** | | **not met**  **n (%)** | |
| **Age groups** | |  | | |  |  |  |  |  |  |  |  | |  |  | |  | |
|  | 15-44 | 168 (98.2) | | | 3 (1.8) | 24 (100.0) | 0 (0.0) | 91 (97.8) | 2 (2.2) | 53 (98.1) | 1 (1.9) | 138 (99.3) | | 1 (0.7) | **474 (98.5)** | | **7 (1.5)** | |
|  | 45-54 | 329 (97.6) | | | 8 (2.4) | 83 (100.0) | 0 (0.0) | 330 (98.5) | 5 (1.5) | 149 (98.0) | 3 (2.0) | 250 (98.0) | | 5 (2.0) | **1,141 (98.2)** | | **21 (1.8)** | |
|  | 55-64 | 490 (99.0) | | | 5 (1.0) | 149 (96.8) | 5 (3.2) | 671 (98.2) | 12 (1.8) | 333 (97.7) | 8 (2.3) | 501 (98.4) | | 8 (1.6) | **2,144 (98.3)** | | **38 (1.7)** | |
|  | 65-74 | 422 (96.6) | | | 15 (3.4) | 136 (95.8) | 6 (4.2) | 814 (97.8) | 18 (2.2) | 484 (98.0) | 10 (2.0) | 639 (98.2) | | 12 (1.8) | **2,495 (97.6)** | | **61 (2.4)** | |
|  | 75+ | 288 (96.6) | | | 10 (3.4) | 110 (97.3) | 3 (2.7) | 566 (98.8) | 7 (1.2) | 415 (97.6) | 10 (2.4) | 673 (96.0) | | 28 (4.0) | **2,052 (97.3)** | | **58 (2.7)** | |
| Deprivation quintile | | | | |  |  |  |  |  |  |  |  | |  |  | |  | |
| 1 - least deprived | | 376 (97.7) | | | 9 (2.3) | 122 (96.8) | 4 (3.2) | 516 (97.5) | 13 (2.5) | 312 (97.2) | 9 (2.8) | 474 (98.5) | | 7 (1.5) | **1,800 (97.7)** | | **42 (2.3)** | |
|  | 2 | 338 (97.1) | | | 10 (2.9) | 124 (96.9) | 4 (3.1) | 584 (98.5) | 9 (1.5) | 365 (98.6) | 5 (1.4) | 511 (97.1) | | 15 (2.9) | **1,922 (97.8)** | | **43 (2.2)** | |
|  | 3 | 383 (98.2) | | | 7 (1.8) | 102 (99.0) | 1 (1.0) | 529 (98.1) | 10 (1.9) | 296 (98.0) | 6 (2.0) | 494 (97.1) | | 15 (2.9) | **1,804 (97.9)** | | **39 (2.1)** | |
|  | 4 | 329 (97.3) | | | 9 (2.7) | 76 (95.0) | 4 (5.0) | 466 (98.1) | 9 (1.9) | 282 (96.6) | 10 (3.4) | 413 (97.4) | | 11 (2.6) | **1,566 (97.3)** | | **43 (2.7)** | |
| 5 - most deprived | | 271 (97.8) | | | 6 (2.2) | 78 (98.7) | 1 (1.3) | 377 (99.2) | 3 (0.8) | 179 (98.9) | 2 (1.1) | 309 (98.1) | | 6 (1.9) | **1,214 (98.5)** | | **18 (1.5)** | |
| **Total** | | **1,697 (97.6)** | | | **41 (2.4)** | **502 (97.3)** | **14 (2.7)** | **2,472 (98.3)** | **44 (1.7)** | **1,434 (97.8)** | **32 (2.2)** | **2,201 (97.6)** | | **54 (2.4)** | **8,306 (97.8)** | | **185 (2.2)** | |
