# Supplementary material for: Can we assess Cancer Waiting Time targets with cancer survival? A population-based study of individually linked data from the National Cancer Waiting Times monitoring dataset in England, 2009-2013
Source: PLoS One. 2018 Aug 22;13(8):e0201288. doi: 10.1371/journal.pone.0201288 (PMC6104918; doi:10.1371/journal.pone.0201288)
Supplement: S3 Table — (DOCX) [file pone.0201288.s008.docx]

**S3 Table: 62-day target attainment by patient characteristics for each cancer site, by stage, England, 2009-13**

| **Colorectal cancer** | | | **Stage I** | | | | | **Stage II** | | | | **Stage III** | | | | **Stage IV** | | | | **Missing** | | | | **All stages** | | |  |  |
| --- | --- | --- | --- | --- | --- | --- | --- | --- | --- | --- | --- | --- | --- | --- | --- | --- | --- | --- | --- | --- | --- | --- | --- | --- | --- | --- | --- | --- |
| **62-day Target attainment** | | | **met**  **n (%)** | **not met**  **n (%)** | | | | **met**  **n (%)** | | **not met**  **n (%)** | | **met**  **n (%)** | | **not met**  **n (%)** | | **met**  **n (%)** | **not met**  **n (%)** | | | **met**  **n (%)** | **not met**  **n (%)** | | | **met**  **n (%)** | **not met**  **n (%)** | |  |  |
| **Age groups** | | |  |  | | | |  | |  | |  | |  | |  |  | | |  |  | | |  |  | |  |  |
| 15-44 | | | 57 (77.0) | 17 (23.0) | | | | 98 (79.7) | | 25 (20.3) | | 138 (78.9) | | 37 (21.1) | | 153 (77.7) | 44 (22.3) | | | 148 (86.0) | 24 (14.0) | | | **594 (80.2)** | **147 (19.8)** | |  |  |
| 45-54 | | | 283 (75.3) | 93 (24.7) | | | | 445 (79.3) | | 116 (20.7) | | 529 (79.2) | | 139 (20.8) | | 655 (78.3) | 182 (21.7) | | | 653 (81.6) | 147 (18.4) | | | **2,565 (79.1)** | **677 (20.9)** | |  |  |
| 55-64 | | | 758 (77.3) | 222 (22.7) | | | | 1,192 (77.6) | | 345 (22.4) | | 1,556 (75.5) | | 504 (24.5) | | 1,584 (77.0) | 472 (23.0) | | | 1,507 (76.4) | 465 (23.6) | | | **6,597 (76.7)** | **2,008 (23.3)** | |  |  |
| 65-74 | | | 1,134 (71.5) | 453 (28.5) | | | | 2,054 (74.3) | | 710 (25.7) | | 2,496 (72.8) | | 931 (27.2) | | 2,336 (74.6) | 794 (25.4) | | | 1,932 (73.4) | 701 (26.6) | | | **9,952 (73.5)** | **3,589 (26.5)** | |  |  |
| 75+ | | | 1,517 (66.2) | 775 (33.8) | | | | 3,512 (71.0) | | 1,437 (29.0) | | 3,973 (70.0) | | 1,702 (30.0) | | 2,950 (68.6) | 1,352 (31.4) | | | 2,494 (74.3) | 861 (25.7) | | | **14,446 (70.2)** | **6,127 (29.8)** | |  |  |
| **Deprivation quintile** | | | | | | | |  | |  | |  | |  | |  |  | | |  |  | | |  |  | |  |  |
| 1 - least deprived | | | 811 (70.1) | 346 (29.9) | | | | 1,617 (73.8) | | 575 (26.2) | | 1,630 (72.8) | | 1,859 (74.1) | | 649 (25.9) | 609 (27.2) | | | 1,414 (74.5) | 483 (25.5) | | | **7,331 (73.4)** | **2,662 (26.6)** | |  |  |
| 2 | | | 881 (70.5) | 369 (29.5) | | | | 1,666 (73.6) | | 597 (26.4) | | 1,901 (72.1) | | 735 (27.9) | | 1,678 (72.2) | 645 (27.8) | | | 1,517 (76.0) | 480 (24.0) | | | **7,643 (73.0)** | **2,826 (27.0)** | |  |  |
| 3 | | | 796 (73.8) | 283 (26.2) | | | | 1,532 (72.5) | | 580 (27.5) | | 1,887 (70.8) | | 778 (29.2) | | 1,631 (72.0) | 634 (28.0) | | | 1,450 (75.4) | 473 (24.6) | | | **7,296 (72.6)** | **2,748 (27.4)** | |  |  |
| 4 | | | 724 (69.3) | 320 (30.7) | | | | 1,423 (75.0) | | 475 (25.0) | | 1,758 (72.5) | | 668 (27.5) | | 1,501 (72.8) | 562 (27.2) | | | 1,326 (74.5) | 455 (25.5) | | | **6,732 (73.1)** | **2,480 (26.9)** | |  |  |
| 5 - most deprived | | | 537 (68.9) | 242 (31.1) | | | | 1,063 (72.4) | | 406 (27.6) | | 1,238 (75.9) | | 1,287 (72.7) | | 483 (27.3) | 394 (24.1) | | | 1,027 (77.0) | 307 (23.0) | | | **5,152 (73.8)** | **1,832 (26.2)** | |  |  |
| **Sex** | | |  |  | | | |  | |  | |  | |  | |  |  | | |  |  | | |  |  | |  |  |
| Female | | | 1,549 (69.7) | 673 (30.3) | | | | 3,191 (72.6) | | 1,206 (27.4) | | 3,922 (72.5) | | 1,485 (27.5) | | 3,169 (72.0) | 1,230 (28.0) | | | 2,743 (76.4) | 847 (23.6) | | | **14,574 (72.8)** | **5,441 (27.2)** | |  |  |
| Male | | | 2,200 (71.3) | 887 (28.7) | | | | 4,110 (74.2) | | 1,427 (25.8) | | 4,770 (72.3) | | 1,828 (27.7) | | 4,509 (73.6) | 1,614 (26.4) | | | 3,991 (74.7) | 1,351 (25.3) | | | **19,580 (73.4)** | **7,107 (26.6)** | |  |  |
| **Tumour site** | | |  |  | | | |  | |  | |  | |  | |  |  | | |  |  | | |  |  | |  |  |
| colon | | | 1,493 (72.4) | 570 (27.6) | | | | 4,643 (74.6) | | 1,583 (25.4) | | 5,124 (73.8) | | 1,820 (26.2) | | 3,935 (75.2) | 1,299 (24.8) | | | 3,814 (75.8) | 1,216 (24.2) | | | **19,009 (74.6)** | **6,488 (25.4)** | |  |  |
| rectum | | | 2,256 (69.5) | 990 (30.5) | | | | 2,658 (71.7) | | 1,050 (28.3) | | 3,568 (70.5) | | 1,493 (29.5) | | 3,743 (70.8) | 1,545 (29.2) | | | 2,920 (74.8) | 982 (25.2) | | | **15,145 (71.4)** | **6,060 (28.6)** | |  |  |
| **Total** | | | **3,749 (70.6)** | **1,560 (29.4)** | | | | **7,301 (73.5)** | | **2,633 (26.5)** | | **7,678 (73.0)** | | **8,692 (72.4)** | | **3,313 (27.6)** | **2,844 (27.0)** | | | **6,734 (75.4)** | **2,198 (24.6)** | | | **34,154 (73.1)** | **12,548 (26.9)** | |  |  |
| **Lung cancer** | |  | | | | |  | | | | |  | | | |  | | |  | | | |  | | | | | |
| **62-day Target attainment** | | | **met**  **n (%)** | **not met**  **n (%)** | | | | **met**  **n (%)** | | **not met**  **n (%)** | | **met**  **n (%)** | | **not met**  **n (%)** | | **met**  **n (%)** | **not met**  **n (%)** | | | **met**  **n (%)** | **not met**  **n (%)** | | | **met**  **n (%)** | **not met**  **n (%)** | |  |  |
| **Age groups** | |  | |  | | |  | | | |  |  |  | | |  |  | |  | |  | |  | |  | | |  |
|  | 15-44 | 28 (63.6) | | 16 (36.4) | | | 27 (84.4) | | | | 5 (15.6) | 75 (77.3) | 22 (22.7) | | | 155 (86.1) | | 25 (13.9) | 23 (76.7) | | 7 (23.3) | | **308 (80.4)** | | **75 (19.6)** | | |  |
|  | 45-54 | 192 (72.5) | | 73 (27.5) | | | 126 (63.6) | | | | 72 (36.4) | 574 (77.3) | 169 (22.7) | | | 869 (85.5) | | 147 (14.5) | 129 (80.1) | | 32 (19.9) | | **1,890 (79.3)** | | **493 (20.7)** | | |  |
|  | 55-64 | 661 (62.0) | | 405 (38.0) | | | 576 (64.8) | | | | 313 (35.2) | 2,141 (74.8) | 722 (25.2) | | | 2,915 (84.5) | | 536 (15.5) | 425 (78.7) | | 115 (21.3) | | **6,718 (76.3)** | | **2,091 (23.7)** | | |  |
|  | 65-74 | 1,335 (59.4) | | 914 (40.6) | | | 906 (57.6) | | | | 668 (42.4) | 3,380 (72.7) | 1,267 (27.3) | | | 4,396 (82.3) | | 943 (17.7) | 599 (74.5) | | 205 (25.5) | | **10,616 (72.6)** | | **3,997 (27.4)** | | |  |
|  | 75+ | 1,375 (59.6) | | 933 (40.4) | | | 938 (62.2) | | | | 569 (37.8) | 3,152 (76.0) | 993 (24.0) | | | 3,960 (84.6) | | 719 (15.4) | 839 (83.6) | | 164 (16.4) | | **10,264 (75.2)** | | **3,378 (24.8)** | | |  |
| **Lung cancer** | | **Stage I** | | | | | **Stage II** | | | | | **Stage III** | | | | **Stage IV** | | | **Missing** | | | | **All stages** | | | | | |
| **62-day Target attainment** | | **met**  **n (%)** | | **not met**  **n (%)** | | | **met**  **n (%)** | | | | **not met**  **n (%)** | **met**  **n (%)** | **not met**  **n (%)** | | | **met**  **n (%)** | **not met**  **n (%)** | | **met**  **n (%)** | | **not met**  **n (%)** | | **met**  **n (%)** | | **not met**  **n (%)** | | |  |
| **Deprivation quintile** | | | |  | | |  | | | |  |  |  | | |  | |  |  | |  | |  | |  | | |  |
| 1 - least deprived | | 498 (63.0) | | 293 (37.0) | | | 372 (63.4) | | | | 215 (36.6) | 1,255 (75.3) | 411 (24.7) | | | 1,913 (84.3) | | 356 (15.7) | 299 (79.3) | | 78 (20.7) | | **4,337 (76.2)** | | **1,353 (23.8)** | | |  |
|  | 2 | 615 (60.3) | | 405 (39.7) | | | 478 (65.0) | | | | 257 (35.0) | 1,636 (75.1) | 542 (24.9) | | | 2,241 (83.6) | | 439 (16.4) | 380 (78.0) | | 107 (22.0) | | **5,350 (75.4)** | | **1,750 (24.6)** | | |  |
|  | 3 | 692 (60.4) | | 453 (39.6) | | | 491 (59.2) | | | | 339 (40.8) | 1,860 (74.4) | 641 (25.6) | | | 2,469 (82.8) | | 512 (17.2) | 402 (81.2) | | 93 (18.8) | | **5,914 (74.4)** | | **2,038 (25.6)** | | |  |
|  | 4 | 835 (58.7) | | 587 (41.3) | | | 594 (60.1) | | | | 394 (39.9) | 2,204 (73.2) | 807 (26.8) | | | 2,822 (84.0) | | 538 (16.0) | 481 (79.9) | | 121 (20.1) | | **6,936 (73.9)** | | **2,447 (26.1)** | | |  |
| 5 - most deprived | | 951 (61.2) | | 603 (38.8) | | | 638 (60.2) | | | | 422 (39.8) | 2,367 (75.4) | 772 (24.6) | | | 2,850 (84.4) | | 525 (15.6) | 453 (78.5) | | 124 (21.5) | | **7,259 (74.8)** | | **2,446 (25.2)** | | |  |
| **Sex** | |  | |  | | |  | | | |  |  |  | | |  | |  |  | |  | |  | |  | | |  |
|  | Female | 1,895 (61.9) | | 1,168 (38.1) | | | 1,126 (63.4) | | | | 651 (36.6) | 4,159 (75.7) | 1,333 (24.3) | | | 5,576 (83.7) | | 1,086 (16.3) | 972 (80.5) | | 235 (19.5) | | **13,728 (75.4)** | | **4,473 (24.6)** | | |  |
|  | Male | 1,696 (59.1) | | 1,173 (40.9) | | | 1,447 (59.7) | | | | 976 (40.3) | 5,163 (73.7) | 1,840 (26.3) | | | 6,719 (84.0) | | 1,284 (16.0) | 1,043 (78.4) | | 288 (21.6) | | **16,068 (74.3)** | | **5,561 (25.7)** | | |  |
| **Total** | | **3,591 (60.5)** | | **2,341 (39.5)** | | | **2,573 (61.3)** | | | | **1,627 (38.7)** | **9,322 (74.6)** | **3,173 (25.4)** | | | **12,295 (83.8)** | **2,370 (16.2)** | | **2,015 (79.4)** | | **523 (20.6)** | | **29,796 (74.8)** | | **10,034 (25.2)** | | |  |
| **Ovarian cancer** | | **Stage I** | | | | **Stage II** | | | | | | **Stage III** | | | | **Stage IV** | | | **Missing** | | | | **All stages** | | | | | |
| **62-day Target attainment** | | **met**  **n (%)** | | | **not met**  **n (%)** | **met**  **n (%)** | | | **not met**  **n (%)** | | | **met**  **n (%)** | | | **not met**  **n (%)** | **met**  **n (%)** | | **not met**  **n (%)** | **met**  **n (%)** | | | **not met**  **n (%)** | **met**  **n (%)** | | | **not met**  **n (%)** | |  |
| **Age groups** | |  | | |  |  | | |  | | |  | | |  |  | |  |  | | |  |  | | |  | |  |
|  | 15-44 | 150 (87.7) | | | 21 (12.3) | 20 (87.0) | | | 3 (13.0) | | | 84 (93.3) | | | 6 (6.7) | 50 (98.0) | | 1 (2.0) | 126 (95.5) | | | 6 (4.5) | **430 (92.1)** | | | **37 (7.9)** | |  |
|  | 45-54 | 310 (92.0) | | | 27 (8.0) | 76 (91.6) | | | 7 (8.4) | | | 298 (89.5) | | | 35 (10.5) | 133 (90.5) | | 14 (9.5) | 219 (89.4) | | | 26 (10.6) | **1,036 (90.5)** | | | **109 (9.5)** | |  |
|  | 55-64 | 447 (90.7) | | | 46 (9.3) | 134 (88.2) | | | 18 (11.8) | | | 584 (87.7) | | | 82 (12.3) | 278 (88.0) | | 38 (12.0) | 416 (87.8) | | | 58 (12.2) | **1,859 (88.5)** | | | **242 (11.5)** | |  |
|  | 65-74 | 377 (87.5) | | | 54 (12.5) | 123 (86.6) | | | 19 (13.4) | | | 671 (84.6) | | | 122 (15.4) | 338 (78.1) | | 95 (21.9) | 484 (84.2) | | | 91 (15.8) | **1,993 (84.0)** | | | **381 (16.0)** | |  |
|  | 75+ | 243 (83.5) | | | 48 (16.5) | 80 (76.2) | | | 25 (23.8) | | | 368 (75.7) | | | 118 (24.3) | 242 (80.7) | | 58 (19.3) | 395 (78.5) | | | 108 (21.5) | **1,328 (78.8)** | | | **357 (21.2)** | |  |
| **Deprivation quintile** | | | | |  |  | | |  | | |  | | |  |  | |  |  | | |  |  | | |  | |  |
| 1 - least deprived | | 335 (87.2) | | | 49 (12.8) | 105 (84.7) | | | 19 (15.3) | | | 426 (84.7) | | | 77 (15.3) | 226 (82.8) | | 47 (17.2) | 339 (81.7) | | | 76 (18.3) | **1,431 (84.2)** | | | **268 (15.8)** | |  |
|  | 2 | 304 (88.6) | | | 39 (11.4) | 109 (87.2) | | | 16 (12.8) | | | 465 (83.5) | | | 92 (16.5) | 277 (87.1) | | 41 (12.9) | 384 (85.5) | | | 65 (14.5) | **1,539 (85.9)** | | | **253 (14.1)** | |  |
|  | 3 | 350 (90.4) | | | 37 (9.6) | 82 (82.0) | | | 18 (18.0) | | | 438 (85.5) | | | 74 (14.5) | 220 (84.9) | | 39 (15.1) | 374 (86.0) | | | 61 (14.0) | **1,464 (86.5)** | | | **229 (13.5)** | |  |
|  | 4 | 299 (89.3) | | | 36 (10.7) | 66 (85.7) | | | 11 (14.3) | | | 376 (85.5) | | | 64 (14.5) | 195 (78.9) | | 52 (21.1) | 320 (88.2) | | | 43 (11.8) | **1,256 (85.9)** | | | **206 (14.1)** | |  |
| 5 - most deprived | | 239 (87.2) | | | 35 (12.8) | 71 (89.9) | | | 8 (10.1) | | | 300 (84.3) | | | 56 (15.7) | 123 (82.0) | | 27 (18.0) | 223 (83.5) | | | 44 (16.5) | **956 (84.9)** | | | **170 (15.1)** | |  |
| **Total** | | **1,527 (88.6)** | | | **196 (11.4)** | **433 (85.7)** | | | **72 (14.3)** | | | **2,005 (84.7)** | | | **363 (15.3)** | **1,041 (83.5)** | | **206 (16.5)** | **1,640 (85.0)** | | | **289 (15.0)** | **6,646 (85.5)** | | | **1,126 (14.5)** | |  |
