# Supplementary material for: Can we assess Cancer Waiting Time targets with cancer survival? A population-based study of individually linked data from the National Cancer Waiting Times monitoring dataset in England, 2009-2013
Source: PLoS One. 2018 Aug 22;13(8):e0201288. doi: 10.1371/journal.pone.0201288 (PMC6104918; doi:10.1371/journal.pone.0201288)
Supplement: S4 Table — (DOCX) [file pone.0201288.s009.docx]

**S4 Table: 31-day target attainment by patient characteristics for each cancer site, by stage, England, 2009-13**

| **Colorectal cancer** | | | **Stage I** | | | **Stage II** | | **Stage III** | | **Stage IV** | | | **Missing** | | | **All stages** | |  |
| --- | --- | --- | --- | --- | --- | --- | --- | --- | --- | --- | --- | --- | --- | --- | --- | --- | --- | --- |
| **31-day Target attainment** | | | **met**  **n (%)** | **not met**  **n (%)** | | **met**  **n (%)** | **not met**  **n (%)** | **met**  **n (%)** | **not met**  **n (%)** | **met**  **n (%)** | **not met**  **n (%)** | | **met**  **n (%)** | **not met**  **n (%)** | | **met**  **n (%)** | **not met**  **n (%)** |  |
| **Age groups** | | |  |  | |  |  |  |  |  |  | |  |  | |  |  |  |
| 15-44 | | | 344 (96.9) | 11 (3.1) | | 567 (98.4) | 9 (1.6) | 823 (98.0) | 17 (2.0) | 783 (98.7) | 10 (1.3) | | 968 (99.0) | 10 (1.0) | | 3,485 (98.4) | 57 (1.6) |  |
| 45-54 | | | 918 (97.3) | 25 (2.7) | | 1,419 (97.5) | 36 (2.5) | 1,946 (98.0) | 40 (2.0) | 1,913 (99.2) | 16 (0.8) | | 1,935 (97.4) | 52 (2.6) | | 8,131 (98.0) | 169 (2.0) |  |
| 55-64 | | | 3,027 (95.9) | 131 (4.1) | | 3,936 (96.9) | 125 (3.1) | 5,121 (96.6) | 181 (3.4) | 4,363 (98.6) | 64 (1.4) | | 6,056 (96.9) | 192 (3.1) | | 22,503 (97.0) | 693 (3.0) |  |
| 65-74 | | | 5,080 (95.9) | 217 (4.1) | | 7,064 (96.4) | 266 (3.6) | 7,657 (96.7) | 265 (3.3) | 5,993 (98.2) | 112 (1.8) | | 9,650 (95.8) | 423 (4.2) | | 35,444 (96.5) | 1,283 (3.5) |  |
| 75+ | | | 4,481 (95.2) | 228 (4.8) | | 9,719 (95.9) | 415 (4.1) | 8,621 (96.2) | 343 (3.8) | 6,874 (98.3) | 120 (1.7) | | 13,043 (96.3) | 508 (3.7) | | 42,738 (96.4) | 1,614 (3.6) |  |
| **Deprivation quintile** | | | | | |  |  |  |  |  |  | |  |  | |  |  |  |
| 1 - least deprived | | | 3,051 (95.6) | 141 (4.4) | | 4,870 (96.6) | 171 (3.4) | 5,113 (96.4) | 191 (3.6) | 4,170 (98.3) | 73 (1.7) | | 6,763 (96.6) | 236 (3.4) | | 23,967 (96.7) | 812 (3.3) |  |
| 2 | | | 3,062 (95.2) | 153 (4.8) | | 5,061 (96.4) | 190 (3.6) | 5,319 (96.2) | 210 (3.8) | 4,349 (98.4) | 71 (1.6) | | 6,995 (96.1) | 281 (3.9) | | 24,786 (96.5) | 905 (3.5) |  |
| 3 | | | 2,963 (96.0) | 123 (4.0) | | 4,787 (96.1) | 195 (3.9) | 5,088 (96.3) | 196 (3.7) | 4,235 (98.3) | 72 (1.7) | | 6,787 (95.8) | 297 (4.2) | | 23,860 (96.4) | 883 (3.6) |  |
| 4 | | | 2,682 (95.6) | 122 (4.4) | | 4,440 (96.0) | 183 (4.0) | 4,720 (97.0) | 144 (3.0) | 3,981 (98.4) | 66 (1.6) | | 6,363 (96.4) | 237 (3.6) | | 22,186 (96.7) | 752 (3.3) |  |
| 5 - most deprived | | | 2,092 (96.6) | 73 (3.4) | | 3,547 (96.9) | 112 (3.1) | 3,928 (97.4) | 105 (2.6) | 3,191 (98.8) | 40 (1.2) | | 4,744 (97.3) | 134 (2.7) | | 17,502 (97.4) | 464 (2.6) |  |
| **Sex** | | |  |  | |  |  |  |  |  |  | |  |  | |  |  |  |
| Female | | | 5,585 (95.7) | 252 (4.3) | | 10,092 (96.6) | 358 (3.4) | 10,260 (96.9) | 332 (3.1) | 8,399 (98.5) | 131 (1.5) | | 14,086 (96.5) | 506 (3.5) | | 48,422 (96.8) | 1,579 (3.2) |  |
| Male | | | 8,265 (95.8) | 360 (4.2) | | 12,613 (96.2) | 493 (3.8) | 13,908 (96.4) | 514 (3.6) | 11,527 (98.4) | 191 (1.6) | | 17,566 (96.3) | 679 (3.7) | | 63,879 (96.6) | 2,237 (3.4) |  |
| **Tumour site** | | |  |  | |  |  |  |  |  |  | |  |  | |  |  |  |
| colon | | | 6,601 (96.0) | 278 (4.0) | | 15,844 (96.5) | 575 (3.5) | 14,270 (96.7) | 492 (3.3) | 12,922 (98.4) | 211 (1.6) | | 20,700 (96.5) | 742 (3.5) | | 70,337 (96.8) | 2,298 (3.2) |  |
| rectum | | | 7,249 (95.6) | 334 (4.4) | | 6,861 (96.1) | 276 (3.9) | 9,898 (96.5) | 354 (3.5) | 7,004 (98.4) | 111 (1.6) | | 10,952 (96.1) | 443 (3.9) | | 41,964 (96.5) | 1,518 (3.5) |  |
| Total | | | 13,850 (95.8) | 612 (4.2) | | 22,705 (96.4) | 851 (3.6) | 24,168 (96.6) | 846 (3.4) | 19,926 (98.4) | 322 (1.6) | | 31,652 (96.4) | 1185 (3.6) | | 112,301 (96.7) | 3,816 (3.3) |  |
| **Lung cancer** | | **Stage I** | | | | **Stage II** | | **Stage III** | | **Stage IV** | | **Missing** | | | **All stages** | | | |
| **31-day Target attainment** | | **met**  **n (%)** | | **not met**  **n (%)** | | **met**  **n (%)** | **not met**  **n (%)** | **met**  **n (%)** | **not met**  **n (%)** | **met**  **n (%)** | **not met**  **n (%)** | **met**  **n (%)** | | **not met**  **n (%)** | **met**  **n (%)** | | **not met**  **n (%)** | |
| **Age groups** | |  | |  | |  |  |  |  |  |  |  | |  |  | |  | |
|  | 15-44 | 149 (88.7) | | 19 (11.3) | | 70 (94.6) | 4 (5.4) | 217 (97.3) | 6 (2.7) | 522 (99.4) | 3 (0.6) | 143 (97.3) | | 4 (2.7) | 1,101 (96.8) | | 36 (3.2) | |
|  | 45-54 | 611 (93.4) | | 43 (6.6) | | 415 (95.6) | 19 (4.4) | 1,385 (97.9) | 29 (2.1) | 2,361 (99.5) | 12 (0.5) | 434 (97.7) | | 10 (2.3) | 5,206 (97.9) | | 113 (2.1) | |
|  | 55-64 | 2,450 (92.5) | | 199 (7.5) | | 1,578 (95.4) | 76 (4.6) | 5,079 (98.3) | 86 (1.7) | 7,356 (99.4) | 48 (0.6) | 1,421 (98.1) | | 28 (1.9) | 17,884 (97.6) | | 437 (2.4) | |
|  | 65-74 | 5,208 (93.3) | | 373 (6.7) | | 2,980 (95.3) | 146 (4.7) | 8,475 (98.5) | 125 (1.5) | 10,900 (99.5) | 54 (0.5) | 2,196 (97.4) | | 58 (2.6) | 29,759 (97.5) | | 756 (2.5) | |
|  | 75+ | 5,985 (94.8) | | 326 (5.2) | | 3,212 (96.6) | 114 (3.4) | 8,153 (98.6) | 112 (1.4) | 10,132 (99.5) | 54 (0.5) | 2,838 (99.0) | | 30 (1.0) | 30,320 (97.9) | | 636 (2.1) | |
| **Lung cancer** | | **Stage I** | | | | **Stage II** | | **Stage III** | | **Stage IV** | | **Missing** | | | **All stages** | | | |
| **31-day Target attainment** | | **met**  **n (%)** | | **not met**  **n (%)** | | **met**  **n (%)** | **not met**  **n (%)** | **met**  **n (%)** | **not met**  **n (%)** | **met**  **n (%)** | **not met**  **n (%)** | **met**  **n (%)** | | **not met**  **n (%)** | **met**  **n (%)** | | **not met**  **n (%)** | |
| **Deprivation quintile** | | | |  | |  |  |  |  |  |  |  | |  |  | |  | |
| 1 - least deprived | | 1,884 (93.1) | | 19 (11.3) | | 70 (94.6) | 4 (5.4) | 3,008 (98.1) | 57 (1.9) | 4,603 (99.3) | 33 (0.7) | 1,081 (98.7) | | 14 (1.3) | 11,702 (97.6) | | 290 (2.4) | |
|  | 2 | 2,419 (93.7) | | 43 (6.6) | | 415 (95.6) | 19 (4.4) | 3,902 (98.7) | 50 (1.3) | 5,589 (99.5) | 29 (0.5) | 1,304 (98.0) | | 27 (2.0) | 14,613 (97.8) | | 324 (2.2) | |
|  | 3 | 2,758 (94.6) | | 199 (7.5) | | 1,578 (95.4) | 76 (4.6) | 4,583 (98.6) | 64 (1.4) | 6,310 (99.5) | 33 (0.5) | 1,387 (97.9) | | 30 (2.1) | 16,657 (97.9) | | 355 (2.1) | |
|  | 4 | 3,343 (93.3) | | 373 (6.7) | | 2,980 (95.3) | 146 (4.7) | 5,556 (98.6) | 79 (1.4) | 7,294 (99.4) | 46 (0.6) | 1,626 (98.6) | | 23 (1.4) | 19,741 (97.7) | | 472 (2.3) | |
| 5 - most deprived | | 3,999 (93.9) | | 326 (5.2) | | 3,212 (96.6) | 114 (3.4) | 6,260 (98.3) | 108 (1.7) | 7,475 (99.6) | 30 (0.4) | 1,634 (97.8) | | 36 (2.2) | 21,557 (97.6) | | 537 (2.4) | |
| **Sex** | |  | |  | |  |  |  |  |  |  |  | |  |  | |  | |
|  | Female | 7,300 (93.7) | | 487 (6.3) | | 3,452 (95.9) | 148 (4.1) | 10,246 (98.5) | 151 (1.5) | 14,343 (99.4) | 88 (0.6) | 3,388 (98.1) | | 67 (1.9) | 38,729 (97.6) | | 941 (2.4) | |
|  | Male | 7,103 (93.8) | | 473 (6.2) | | 4,803 (95.8) | 211 (4.2) | 13,063 (98.4) | 207 (1.6) | 16,928 (99.5) | 83 (0.5) | 3,644 (98.3) | | 63 (1.7) | 45,541 (97.8) | | 1,037 (2.2) | |
| **Total** | | 14,403 (93.8) | | 960 (6.2) | | 8,255 (95.8) | 359 (4.2) | 23,309 (98.5) | 358 (1.5) | 31,271 (99.5) | 171 (0.5) | 7,032 (98.2) | | 130 (1.8) | 84,270 (97.7) | | 1,978 (2.3) | |
| **Ovarian cancer** | | **Stage I** | | | | **Stage II** | | **Stage III** | | **Stage IV** | | **Missing** | | | **All stages** | | | |
| **31-day Target attainment** | | **met**  **n (%)** | | | **not met**  **n (%)** | **met**  **n (%)** | **not met**  **n (%)** | **met**  **n (%)** | **not met**  **n (%)** | **met**  **n (%)** | **not met**  **n (%)** | **met**  **n (%)** | | **not met**  **n (%)** | **met**  **n (%)** | | **not met**  **n (%)** | |
| **Age groups** | |  | | |  |  |  |  |  |  |  |  | |  |  | |  | |
|  | 15-44 | 450 (98.0) | | | 9 (2.0) | 72 (97.3) | 2 (2.7) | 242 (98.8) | 3 (1.2) | 126 (100.0) | 0 (0.0) | 361 (98.1) | | 7 (1.9) | 1,251 (98.3) | | 21 (1.7) | |
|  | 45-54 | 634 (97.1) | | | 19 (2.9) | 166 (97.6) | 4 (2.4) | 609 (98.9) | 7 (1.1) | 327 (99.7) | 1 (0.3) | 566 (98.1) | | 11 (1.9) | 2,302 (98.2) | | 42 (1.8) | |
|  | 55-64 | 810 (95.9) | | | 35 (4.1) | 244 (96.4) | 9 (3.6) | 1,166 (98.4) | 19 (1.6) | 639 (99.7) | 2 (0.3) | 999 (98.4) | | 16 (1.6) | 3,858 (97.9) | | 81 (2.1) | |
|  | 65-74 | 696 (96.7) | | | 24 (3.3) | 237 (97.9) | 5 (2.1) | 1,433 (98.8) | 17 (1.2) | 871 (99.1) | 8 (0.9) | 1,291 (98.8) | | 16 (1.2) | 4,528 (98.5) | | 70 (1.5) | |
|  | 75+ | 470 (96.1) | | | 19 (3.9) | 185 (96.4) | 7 (3.6) | 894 (98.8) | 11 (1.2) | 620 (99.2) | 5 (0.8) | 1,116 (98.7) | | 15 (1.3) | 3,285 (98.3) | | 57 (1.7) | |
| **Deprivation quintile** | | | | |  |  |  |  |  |  |  |  | |  |  | |  | |
| 1 - least deprived | | 651 (95.9) | | | 28 (4.1) | 206 (96.3) | 8 (3.7) | 912 (98.7) | 12 (1.3) | 526 (98.9) | 6 (1.1) | 932 (98.8) | | 11 (1.2) | 3,227 (98.0) | | 65 (2.0) | |
|  | 2 | 597 (96.6) | | | 21 (3.4) | 217 (97.3) | 6 (2.7) | 1,010 (99.0) | 10 (1.0) | 633 (99.2) | 5 (0.8) | 927 (98.7) | | 12 (1.3) | 3,384 (98.4) | | 54 (1.6) | |
|  | 3 | 669 (96.7) | | | 23 (3.3) | 167 (97.1) | 5 (2.9) | 945 (98.4) | 15 (1.6) | 520 (99.6) | 2 (0.4) | 926 (98.4) | | 15 (1.6) | 3,227 (98.2) | | 60 (1.8) | |
|  | 4 | 608 (97.9) | | | 13 (2.1) | 160 (97.6) | 4 (2.4) | 822 (98.7) | 11 (1.3) | 545 (99.6) | 2 (0.4) | 877 (98.3) | | 15 (1.7) | 3,012 (98.5) | | 45 (1.5) | |
| 5 - most deprived | | 535 (96.2) | | | 21 (3.8) | 154 (97.5) | 4 (2.5) | 655 (98.6) | 9 (1.4) | 359 (99.7) | 1 (0.3) | 671 (98.2) | | 12 (1.8) | 2,374 (98.1) | | 47 (1.9) | |
| **Total** | | 3,060 (96.7) | | | 106 (3.3) | 904 (97.1) | 27 (2.9) | 4,344 (98.7) | 57 (1.3) | 2,583 (99.4) | 16 (0.6) | 4,333 (98.5) | | 65 (1.5) | 15,224 (98.3) | | 271 (1.7) | |
